# Gulp1 controls Eph/ephrin trogocytosis and is important for cell rearrangements during development

**DOI:** 10.1083/jcb.201901032

**Published:** 2019-08-13

**Authors:** Jingyi Gong, Thomas N. Gaitanos, Olivia Luu, Yunyun Huang, Louise Gaitanos, Jana Lindner, Rudolf Winklbauer, Rüdiger Klein

**Affiliations:** 1 Max Planck Institute of Neurobiology, Department of Molecules-Signaling-Development, Munich-Martinsried, Germany; 2 Department of Cell and Systems Biology, University of Toronto, Toronto, Canada

## Abstract

Trogocytosis, in which cells nibble away parts of neighboring cells, is an intercellular cannibalism process conserved from protozoa to mammals. Its underlying molecular mechanisms are not well understood and are likely distinct from phagocytosis, a process that clears entire cells. Bi-directional contact repulsion induced by Eph/ephrin signaling involves transfer of membrane patches and full-length Eph/ephrin protein complexes between opposing cells, resembling trogocytosis. Here, we show that the phagocytic adaptor protein Gulp1 regulates EphB/ephrinB trogocytosis to achieve efficient cell rearrangements of cultured cells and during embryonic development. Gulp1 mediates trogocytosis bi-directionally by dynamic engagement with EphB/ephrinB protein clusters in cooperation with the Rac-specific guanine nucleotide exchange factor Tiam2. Ultimately, Gulp1’s presence at the Eph/ephrin cluster is a prerequisite for recruiting the endocytic GTPase dynamin. These results suggest that EphB/ephrinB trogocytosis, unlike other trogocytosis events, uses a phagocytosis-like mechanism to achieve efficient membrane scission and engulfment.

## Abstract (precis)

Trogocytosis, intercellular cannibalism distinct from phagocytosis, occurs when cells rearrange during development. Here, Gong et al. reveal that trogocytosis induced by ephrins and Eph receptors involves phagocytic adaptor protein Gulp1, Rac-specific guanine nucleotide exchange factor Tiam2, and endocytic GTPase dynamin. These results suggest that ephrin/Eph-induced trogocytosis uses phagocytosis-like mechanisms.

## Introduction

Multicellular organisms often go through processes to clear unwanted or excess cells. Removal of whole dying cells by phagocytosis is evolutionarily conserved and relatively well described (Flannagan et al., 2012; Freeman and Grinstein, 2014; Arandjelovic and Ravichandran, 2015; Lim et al., 2017). In contrast to the removal of entire cell corpses, there are emerging examples in which cells nibble away parts of neighboring cells in a process termed “trogocytosis,” or cell cannibalism, that is less well understood. Available evidence suggests that both common and distinct machineries are engaged in these two processes (Joly and Hudrisier, 2003; Ralston, 2015). Examples of trogocytosis include intercellular transfer of proteins and membrane patches between primordial germ cells (PGCs) and endodermal cells in *Caenorhabditis elegans* (Abdu et al., 2016), between antigen-presenting cells and lymphocytes (Huang et al., 1999; Dopfer et al., 2011), and between neurons and microglia in mice (Weinhard et al., 2018). Partial eating of host cells by amoebae, a process that contributes to cell killing and tissue invasion, has been proposed to be an ancient form of trogocytosis (Ralston et al., 2014; Ralston, 2015).

Cell nibbling also occurs during embryogenesis when cells repel each other after direct cell–cell contact. This partial eating behavior is required to remove the adhesive receptor–ligand complex that forms at the interface of the two opposing cells (Riccomagno and Kolodkin, 2015; Wen and Winklbauer, 2017). Ephrin receptor (Eph) tyrosine kinases and their membrane-bound ephrin ligands are prominent inducers of contact repulsion during embryonic development (Batlle and Wilkinson, 2012; Ventrella et al., 2017). Both receptors and ligands comprise two subfamilies: EphAs that preferentially bind glycosylphosphatidylinositol-anchored ephrinAs and EphBs that prefer binding transmembrane ephrinBs (Kania and Klein, 2016). Ephs and ephrins function in opposing cells, such that ephrins act as trans ligands of Eph receptors, resulting in Eph “forward” signaling and the transfer of the intact ephrin/Eph complex into the Eph-expressing cell. The ephrin is thereby trans-endocytosed into the Eph cell. This process can also happen in the opposite direction—Ephs acting as ligands for ephrins, termed “reverse” signaling, and trans-endocytosis of Ephs into the ephrin cell (Marston et al., 2003; Zimmer et al., 2003; Lauterbach and Klein, 2006). This trans-endocytosis resembles trogocytosis, as intact membrane proteins are being transferred between the cells (Ralston, 2015).

Eph/ephrin-mediated cell repulsion has been intensely studied during embryonic development as a mechanism to sort and position mixed cell populations, set up tissue boundaries, and guide migrating cells and axons (Cayuso et al., 2015; Kania and Klein, 2016). Eph/ephrin signaling also contributes to the migratory behavior and invasiveness of cancer cells (Astin et al., 2010; Batlle and Wilkinson, 2012; Lisabeth et al., 2013; Taylor et al., 2017). Ephrin reverse signaling was recently implicated in the *Xenopus laevis* gastrula, where endodermal cells display amoeboid-like cell migration (Wen and Winklbauer, 2017). Moreover, it was shown for the first time that cell migration in vivo requires resorption of the migrating cell’s tail in part by ephrinB1-dependent trans-endocytosis/trogocytosis (Wen and Winklbauer, 2017).

The underlying molecular mechanisms of trogocytosis, in general, and of Eph/ephrin-driven trogocytosis, in particular, are only beginning to be unraveled. In contrast, phagocytosis has been studied extensively in various model organisms and cell types (Flannagan et al., 2012; Freeman and Grinstein, 2014). Genetic studies in *C. elegans* have highlighted two independent and partially redundant phagocytic pathways for apoptotic cell clearance. One pathway uses CrkII (ced-2), Dock180 (ced-5), and Elmo1 (ced-12) to activate Rac1 (ced-10), while the second route signals through the transmembrane receptor MEGF10 (ced-1), which activates dynamin or actin polymerization via the engulfment adaptor Gulp1 (ced-6; Liu and Hengartner, 1998; Kinchen et al., 2005). Both pathways lead to reorganization of the cytoskeleton to initiate engulfment of the target cell. Whether these two pathways are conserved in mediating trogocytosis, especially Eph/ephrin trogocytosis, has not yet been studied, and it remains unclear to what extent trogocytosis and phagocytosis share common mechanisms (Ralston, 2015). On the one hand, both trogocytosis and phagocytosis depend on precise control of phosphoinositide turnover and cytoskeleton dynamics, which requires phosphoinositide 3-kinase and Rac GTPase activity, respectively (Ralston, 2015). Moreover, activation of small GTPases to promote actin polymerization has been shown to be important for T cell trogocytosis and EphB/ephrinB trogocytosis (Martínez-Martín et al., 2011; Gaitanos et al., 2016). EphB/ephrinB trogocytosis and contact repulsion have further been shown to depend on the recruitment of the Rac-specific guanine nucleotide exchange factor Tiam2 (Gaitanos et al., 2016). On the other hand, distinct players, such as AGC family kinase 1, were recently shown to participate in trogocytosis but not phagocytosis in *Entamoeba histolytica* (Somlata et al., 2017). Moreover, instead of using classical apoptotic engulfment pathways, PGC trogocytosis in *C. elegans* uses lst-4/SNX9, a sorting nexin family member, to recruit dynamin to pinch off PGC protrusions (Abdu et al., 2016).

Here, we investigate the role of the engulfment adaptor Gulp1 (ced-6) in the context of EphB/ephrinB trogocytosis. We show that the Gulp1 protein is dynamically recruited to EphB/ephrinB clusters at the interface of two opposing cells, where it regulates EphB/ephrinB trogocytosis in both forward and reverse directions. As a consequence, Gulp1 is required to achieve efficient cell rearrangements of cultured cells and during *Xenopus* gastrulation. Gulp1 functions in cooperation with the Rac-specific guanine nucleotide exchange factor Tiam2 and by recruiting the endocytic GTPase dynamin to EphB/ephrinB clusters. These results suggest that EphB/ephrinB trogocytosis, unlike other trogocytosis events, utilizes a phagocytosis-like mechanism to achieve efficient membrane scission and engulfment.

## Results

### Gulp1 interacts with EphB2 and ephrinB1 during Eph/ephrin trogocytosis

**Figure 1.**
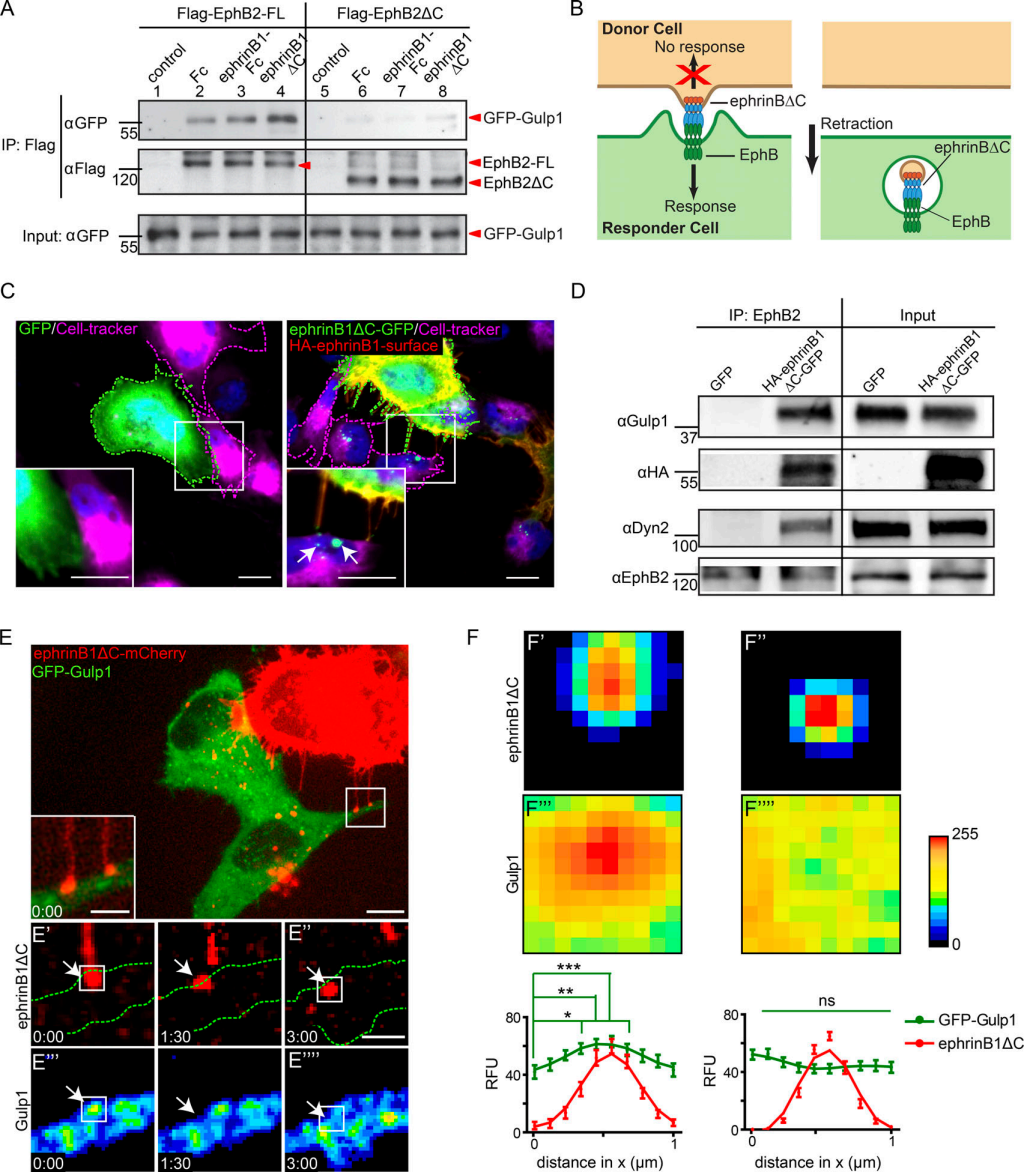
**Gulp1 interacts with EphB2. (A)** Validation of the interaction between Gulp1 and EphB2 using coimmunoprecipitation. HeLa cells overexpressing GFP-Gulp1 and either full-length Flag-EphB2 (Flag-EphB2-FL) or Flag-EphB2ΔC were either not treated (control) or treated with either preclustered Fc or preclustered ephrinB1-Fc, or cocultured with HeLa cells overexpressing ephrinB1ΔC-CFP (ephrinB1ΔC). **(B)** Model of EphB/ephrinB forward trogocytosis. EphrinBΔC from donor cells is trans-endocytosed into EphB^+^ responder cells. **(C)** Representative images showing forward trogocytosis in U251 cells (magenta dashed line, labeled with Cell-tracker) cocultured with ephrinB1ΔC-GFP^+^ donor HeLa cells (right panels, green dashed line), but not control GFP^+^ cells (left panels, green dashed line). Internalized ephrinB1ΔC vesicles in U251 cells were detected as green puncta void of surface HA-antibody labeling (arrows). Scale bars, 10 µm. **(D)** Validation of the interaction between endogenous Gulp1 and EphB2. U251 cells were first cocultured with either control GFP^+^ or ephrinB1ΔC-GFP^+^ HeLa cells for 30 min, and cell lysates were then subjected to immunoprecipitation by anti-EphB2 antibodies. **(E)** Representative images from live imaging of forward trogocytosis in HeLa cells. EphrinB1ΔC-mCherry^+^ donor cells were cocultured with responder cells expressing untagged EphB2 and GFP-Gulp1. Middle row: green dashed lines indicate the responder cell outline. Bottom rows show time course at time of contact and scission. Arrows indicate ephrinB1ΔC cluster formation and subsequent vesicle internalization. GFP-Gulp1 images pseudo-colored as heat maps (bottom row). Scale bars, 10 µm (top panel), 5 µm (inset), and 2 µm (time-lapse images). Elapsed time shown as min:s. **(F)** Average fluorescent intensities at contact sites of cluster formation (F′) and subsequent vesicles (F″) from time-lapse imaging of cocultures as described in E. Data displayed as heat maps of average intensity calculated over every event, with donor contact sites for each set aligned to top and center, and all images normalized to their respective background signal. Graphs show mean ± SEM of relative fluorescent units (RFU) changes through the central four pixels for the x axis. Data acquired from 32 events from 10 cells over two independent experiments. *, P < 0.05; **, P < 0.01; ***, P < 0.001; ns, not significant; one-way ANOVA with Bonferroni’s multiple comparisons test performed on GFP-Gulp1 signal.

We previously identified Gulp1 in a proteomic screen as an interactor of clustered EphB2 (Gong et al., 2016). Gulp1 was highly enriched in the interactome of clustered full-length EphB2, but not of the variant lacking the cytoplasmic domain (EphB2ΔC). To validate this interaction, we performed coimmunoprecipitation experiments in HeLa cells coexpressing a GFP-Gulp1 fusion protein and either full-length EphB2 or EphB2ΔC (both Flag-tagged). Under basal conditions, GFP-Gulp1 was weakly coimmunoprecipitated by full-length EphB2 but not EphB2ΔC (Fig. 1 A, compare lanes 2 and 6). To analyze whether the interaction of Gulp1 and EphB2 was enhanced by ephrinB1 stimulation, we used a cell–cell assay (Fig. 1 B). Forward trogocytosis and uni-directional signaling into the EphB2-expressing cell was achieved by coculturing donor cells expressing C-terminally truncated ephrinB1ΔC with responder cells expressing full-length EphB2 in the presence or absence of GFP-Gulp1 (Video 1; Zimmer et al., 2003; Gaitanos et al., 2016). Trogocytosis under these conditions was dependent on trans interactions of ephrinB1 and EphB2, and on EphB2 forward signaling (Videos 2, 3, and 4). Gulp1 interaction with EphB2-FL was greatly enhanced in the forward trogocytosis condition, but not when responder cells expressed EphB2ΔC (Fig. 1 A, compare lanes 4 and 8; and Fig. S1 A). Interestingly, enhanced interaction of Gulp1 and EphB2 was only seen in the cell–cell stimulation assay but not after stimulation of EphB2-expressing cells with preclustered soluble ephrinB1-Fc (Fig. 1 A, compare lanes 2 and 3; and Fig. S1 A). We further validated the interaction between Gulp1 and EphB2 using U251 glioblastoma cells that endogenously express both proteins. When U251 responder cells were cocultured with ephrinB1ΔC-GFP^+^ donor HeLa cells, forward trogocytosis was observed (Fig. 1 C), and endogenous Gulp1 was coprecipitated with endogenous EphB2 (Fig. 1 D). These results show that the interaction between Gulp1 and EphB2 is specifically strengthened under Eph/ephrin trogocytosis conditions.

Coimmunofluorescence imaging revealed that GFP-Gulp1 localized at ephrinB1ΔC surface clusters in forward trogocytosis situations (Fig. S1 B). To visualize the dynamics of Gulp1 colocalization with ephrinB1 clusters, we performed live imaging of the trogocytosis process (Fig. 1, E and F). Upon contact of the ephrinB1ΔC-mCherry^+^ donor cell (red) with the EphB2-FL^+^ responder cell (green), ephrinB1ΔC-mCherry formed clusters at the EphB2-FL^+^ cell surface (Fig. 1 E′). Subsequently, ephrinB1ΔC-mCherry^+^ vesicles were pinched off from the original cluster and internalized into the responder cell (Fig. 1 E″). GFP-Gulp1 was transiently but significantly enriched at the site of ephrinB1ΔC-mCherry clusters (Fig. 1, E′′′, F, and F′′′). Gulp1 was no longer enriched at ephrinB1 clusters once the vesicles were pinched off and internalized into the responder cell (Fig. 1, E′′′′, F, and F′′′′), suggesting the interaction of Gulp1 and Eph-ephrin clusters was transient. Unlike GFP-Gulp1, GFP control protein did not enrich at the site of ephrinB1ΔC-mCherry clusters (Fig. S1, C and D).

Since EphB/ephrinB trogocytosis often happens bi-directionally, i.e., into both EphB (forward) and ephrinB cells (reverse trogocytosis; Zimmer et al., 2003; Gaitanos et al., 2016), we also asked whether Gulp1 could interact with ephrinB1. When SKN neuroblastoma cells, endogenously expressing ephrinBs, were cocultured with EphB2ΔC-GFP^+^ HeLa donor cells, reverse trogocytosis was observed (Fig. S1 E) and endogenous Gulp1 was coimmunoprecipitated with endogenous ephrinBs (Fig. S1 F). Live imaging (data not shown) revealed that GFP-Gulp1 was localized at the site of EphB2ΔC-mCherry clusters in a reverse trogocytosis cell–cell stimulation assay using ephrinB1-FL^+^ responder cells cocultured with EphB2ΔC-mCherry^+^ donor cells (Fig. S1 G). Together, these results illustrate that Gulp1 is recruited to EphB2 and ephrinB1 clusters during the initial phases of forward and reverse trogocytosis, respectively.

### Gulp1 is required for bi-directional EphB2/ephrinB1 trogocytosis

**Figure 2.**
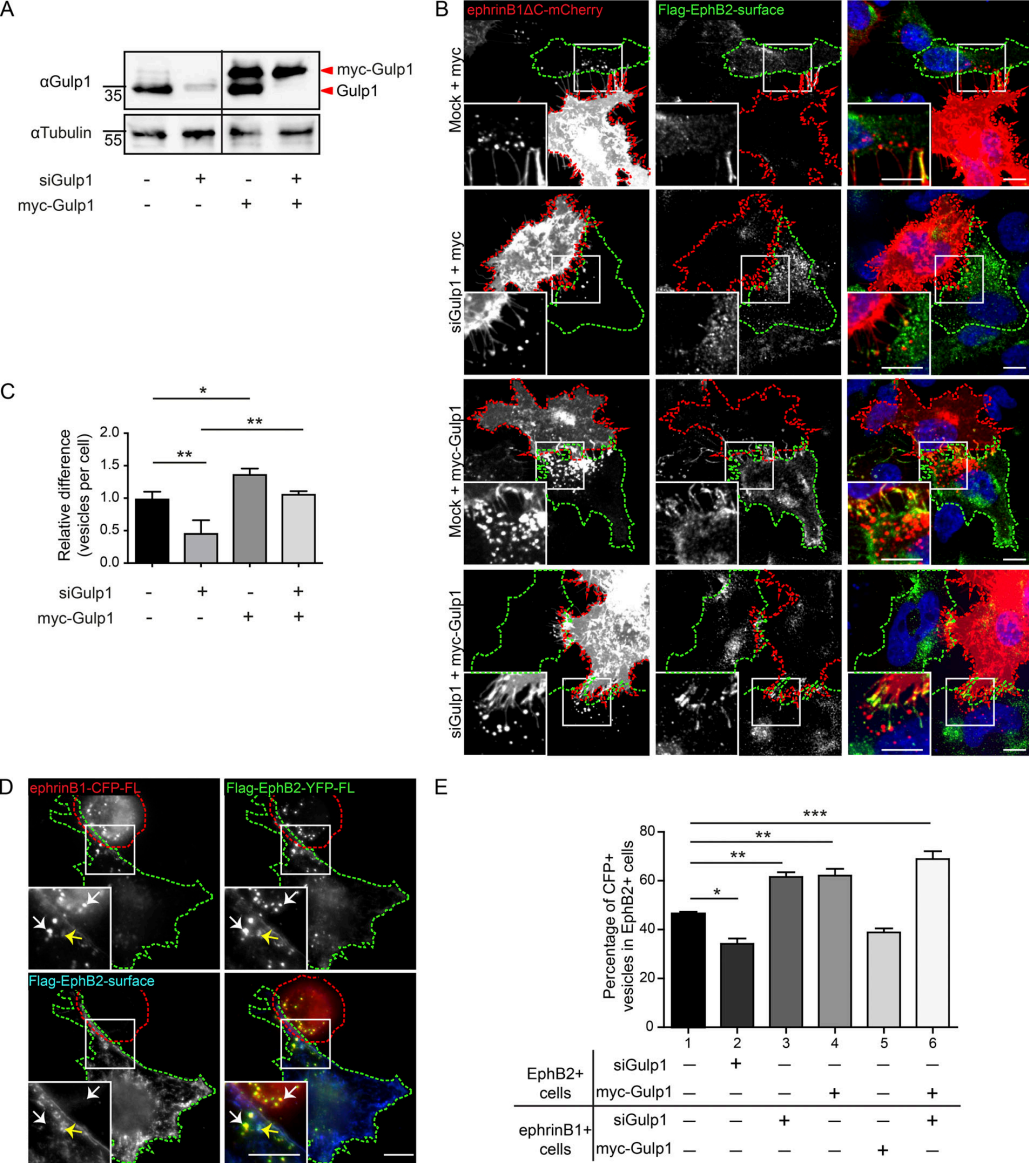
**Gulp1 regulates EphB2/ephrinB1 trogocytosis. (A)** Western blot analysis shows efficient knockdown of endogenous Gulp1 expression and overexpression of siRNA-resistant myc-Gulp1 in HeLa cells. **(B and C)** Representative images (B) and quantification (C) showing Gulp1 is required for forward trogocytosis in HeLa cells. Responder cells (green dashed line) were treated with either mock or Gulp1 siRNA, then overexpressed with Flag-EphB2 and either siRNA-resistant myc-Gulp1 or myc as a control, before coculture with ephrinB1ΔC-mCherry^+^ donor cells (red dashed line). To determine internalized vesicles (red puncta), surface clusters (yellow puncta) were detected by antibody staining against Flag-EphB2 without permeabilization. Scale bars, 10 µm. Quantification results shown as mean ± SEM (*n* = 3 independent experiments, 6–12 responder cells per condition per experiment). Data normalized to median mock value per experiment. *, P < 0.05; **, P < 0.01; one-way ANOVA with Tukey’s multiple comparisons test. **(D and E)** Representative images (D) and quantification (E) showing manipulation of Gulp1 expression levels changes the preference of bi-directional EphB2/ephrinB1 trogocytosis in HeLa cells. Bi-directional trogocytosis was induced by coculturing Flag-EphB2-YFP^+^ cells (green dashed line) with ephrinB1-CFP^+^ cells (red dashed line). Combinations of Gulp1 knockdown and Gulp1 overexpression were set up as shown in E. White arrows indicate internalized vesicles (yellow puncta), and yellow arrows indicate surface clusters (blue puncta). Scale bars, 10 µm. The total pool of internalized YFP^+^/CFP^+^ vesicles within two opposing cells was counted, and the percentage of vesicles in EphB2^+^ cells (forward trogocytosis) was shown in the figure. The percentage of vesicles in ephrinB1^+^ cells (reverse trogocytosis) would be 100% minus the percentage of vesicles in EphB2^+^ cells (not shown). Results shown as mean ± SEM (*n* = 3 independent experiments, 6–11 responder-donor pairs per condition per experiment). *, P < 0.05; **, P < 0.01; ***, P < 0.001; one-way ANOVA with Tukey’s multiple comparisons test.

Next, we asked whether Gulp1 plays a role in EphB2/ephrinB1 trogocytosis by gain- and loss-of-function manipulations. As confirmed by Western blot, Gulp1 can be efficiently knocked down in HeLa cells using stealth siRNA. Expression can then be restored by overexpressing siRNA-resistant Gulp1 (myc-tagged) in Gulp1 knockdown cells (Fig. 2 A). Using the cell–cell stimulation assay described above, we found that decreased levels of Gulp1 in EphB2-FL^+^ responder cells significantly reduced ephrinB1 trans-endocytosis from ephrinB1ΔC^+^ donor cells into EphB2-FL^+^ cells (forward trogocytosis; Fig. 2, B and C). Restoration of Gulp1 expression by overexpressing siRNA-resistant myc-Gulp1 completely rescued ephrinB1 trans-endocytosis. Furthermore, overexpression of myc-Gulp1 in the presence of endogenous Gulp1 was sufficient to increase ephrinB1 trans-endocytosis. Together, these results illustrate that Gulp1 regulates forward trogocytosis.

To test whether Gulp1 is required for bi-directional EphB2/ephrinB1 trogocytosis, full-length ephrinB1-CFP^+^ cells were cocultured with full-length EphB2-YFP^+^ cells (Fig. 2 D). In this tug-of-war situation, trogocytosis happens bi-directionally, with ephrinB1 being internalized into EphB2^+^ cells (forward trogocytosis) and EphB2 being internalized into ephrinB1^+^ cells (reverse trogocytosis) simultaneously. In the control condition where Gulp1 levels were not manipulated, about half (47 ± 1%) of the EphB2/ephrinB1-containing vesicles were trogocytosed into EphB2^+^ cells, while the other half of the vesicles were trogocytosed into ephrinB1^+^ cells (Fig. 2 E). When silencing Gulp1 in EphB2^+^ cells, the percentage of EphB2/ephrinB1-containing vesicles in EphB2^+^ cells decreased to 34 ± 4%, confirming that Gulp1 is necessary for efficient forward trogocytosis. Reciprocally, when Gulp1 was depleted in ephrinB1^+^ cells, reverse trogocytosis was reduced and forward trogocytosis was enhanced in EphB2^+^ cells (62 ± 3%), suggesting that Gulp1 is also required for reverse trogocytosis. Furthermore, by overexpressing Gulp1 in either the EphB2^+^ or ephrinB1^+^ cells, we were able to positively influence the direction of trogocytosis into the cells with higher Gulp1 levels. Finally, overexpressing Gulp1 in the EphB2^+^ cells while simultaneously depleting Gulp1 in the ephrinB1^+^ cells further increased forward trogocytosis in the EphB2^+^ cells (69 ± 5%). These results demonstrate that Gulp1 facilitates bi-directional EphB2/ephrinB1 trogocytosis, and that its relative levels can influence the direction of the response.

### Gulp1 phosphotyrosine-binding (PTB) domain mediates interaction with EphB2

**Figure 3.**
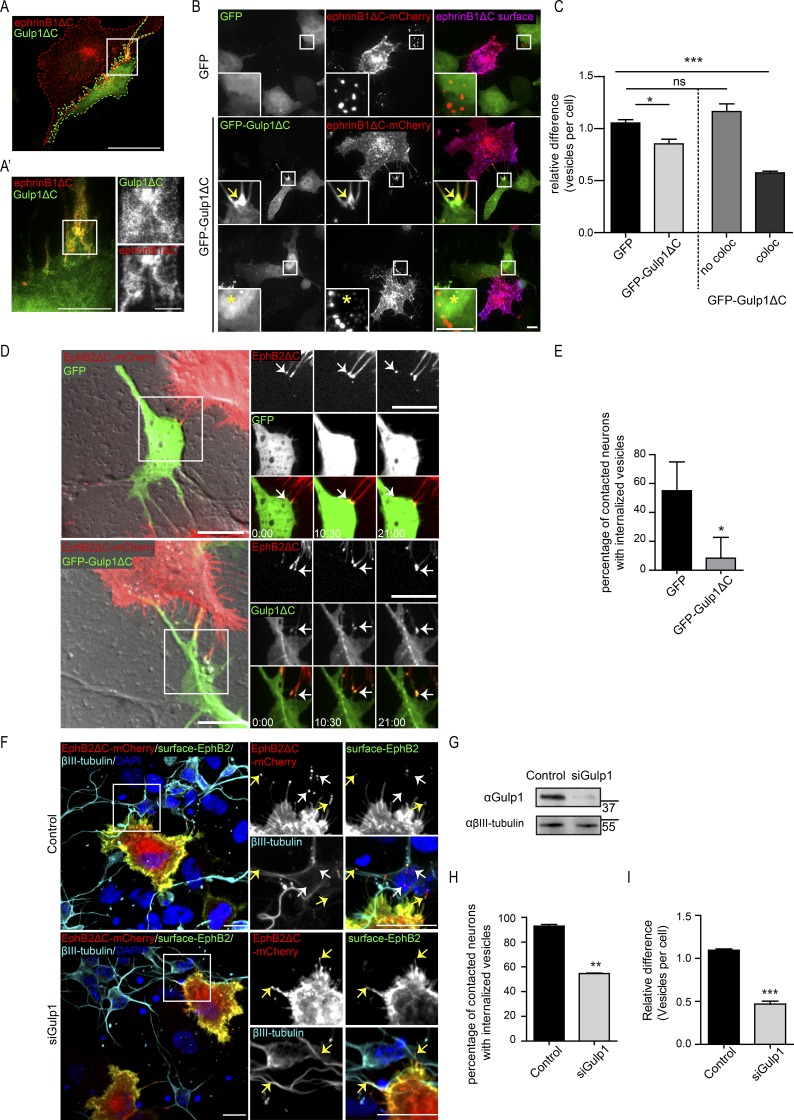
**Gulp1ΔC serves as a dominant-negative protein blocking EphB2/ephrinB1 trogocytosis. (A and A′)** Representative confocal image (A) and STED image (A′) of the same cell showing GFP-Gulp1ΔC specifically colocalizes with EphB2/ephrinB1 clusters (visualized by ephrinB1ΔC-mCherry signal, x-y resolution = 20 nm). Scale bars, 20 µm (A), 5 µm (A′, left panel), and 2 µm (A′, right panels). **(B and C)** Representative images (B) and quantification (C) showing Gulp1ΔC blocks forward trogocytosis in HeLa cells when enriched at EphB2/ephrinB1 clusters. Internalized vesicles detected by comparing total ephrinB1ΔC signal to surface signal. Arrows indicate GFP-Gulp1ΔC enrichment at ephrinB1ΔC clusters; asterisks indicate higher GFP-Gulp1ΔC signal in nucleus. Scale bars, 10 µm. Relative value of vesicles number per cell shown as mean ± SEM (*n* = 4 independent experiments, 25–40 responder cells per condition per experiment). Data normalized to median GFP value per experiment. The right two columns show the GFP-Gulp1ΔC condition separated into cells that either show GFP-Gulp1ΔC and ephrinB1ΔC colocalization (coloc), or not (no coloc). *, P < 0.05; ***, P < 0.001; ns, not significant; one-way ANOVA with Dunnett’s post hoc test. **(D and E)** Representative time-lapse images (D) and quantification (E) showing Gulp1ΔC blocks reverse trogocytosis in cultured cortical neurons. Scale bars, 10 µm. Elapsed time shown as min:s. Arrows indicate EphB2ΔC clusters or subsequent trogocytosed vesicles. Relative percentage of contacted neurons (indicated by EphB2 clusters) with internalized EphB2ΔC vesicles shown as mean ± SEM (*n* = 3 independent experiments, 4–13 neurons per condition per experiment). *, P = 0.0305, two-tailed unpaired *t* test. **(F)** Representative images showing Gulp1 is required for reverse trogocytosis in cultured cortical neurons. Neurons were labeled with βIII-tubulin antibodies. Internalized EphB2ΔC vesicles, indicated by white arrows (red puncta), were differentiated from surface EphB2ΔC clusters, indicated by yellow arrows (yellow puncta). Scale bars, 10 µm. **(G)** Western blot analysis showing efficient knockdown of endogenous Gulp1 expression in primary cultured neurons. **(H)** Quantification showing percentage of contacted neurons (indicated by EphB2 clusters) with internalized EphB2ΔC vesicles. **, P = 0.0015; two-tailed unpaired *t* test. **(I)** Quantification showing relative value of vesicles number per contacted neuron. Data normalized to median control value per experiment. Results shown as mean ± SEM (*n* = 3 independent experiments, 22–29 responder cells per condition per experiment). ***, P = 0.0009; two-tailed unpaired *t* test.

To map the region of Gulp1 that mediates its interaction with EphB2, we generated truncated Gulp1 constructs according to previously described functional domains (Su et al., 2002; Fig. S2 A). We generated GFP fusion constructs consisting of combinations of the N-terminal PTB domain, the zip-finger domain, and a C-terminal leucine-rich region that is presumed to be the effector domain. By performing coimmunoprecipitation assays from transfected cell lysates, we found only constructs containing the PTB domain bound EphB2, demonstrating that the PTB domain is essential for the interaction of Gulp1 with EphB2 (Fig. S2 B). The zip-finger domain (construct N2) further enhanced the interaction of the PTB domain with EphB2. Multi-color stimulated emission depletion microscopy (STED) imaging revealed that this N2 construct (hereafter referred to as Gulp1ΔC) highly and specifically colocalized with ephrinB1ΔC clusters in the responding cell after coculture (Fig. 3, A and A′). The identical localization of Gulp1ΔC and ephrinB1ΔC in the EphB2^+^ cell supports the finding that Gulp1 directly interacts with the Eph/ephrin complex upon cell–cell contact.

Because Gulp1ΔC lacks its putative effector domain and has high affinity to EphB2, we hypothesized that it may have a dominant-negative effect on EphB/ephrinB trogocytosis. When we overexpressed Gulp1ΔC in EphB2-FL^+^ responder cells, we noticed that Gulp1ΔC was strongly enriched at ephrinB1ΔC clusters upon coculture stimulation. This colocalization occurred in only half of the transfected cells (56% ± 4%), whereas in the other half of the cells, Gulp1ΔC was diffusely expressed in the cytoplasm and often enriched in the nucleus (Fig. 3 B). These results raise the possibility that the status of HeLa cells is heterogeneous and that a large fraction expresses other, possibly nuclear, binding partners of Gulp1ΔC. Quantification revealed that Gulp1ΔC expression modestly reduced ephrinB1ΔC trans-endocytosis (forward trogocytosis) when considering all responder cells. However, the inhibitory effect of Gulp1ΔC expression was much stronger in cells with Gulp1ΔC enrichment at ephrinB1ΔC clusters (Fig. 3, B and C; and Fig. S2, C–E). We observed similar inhibitory effects of Gulp1ΔC in the reciprocal direction, i.e., EphB2ΔC trans-endocytosis (reverse trogocytosis; Fig. S2, F and G). To exclude the possibility that expression of Gulp1ΔC leads to a global dysfunction of the endolysosomal system, we induced receptor endocytosis with soluble ephrinB1-Fc, a process mediated by mechanisms distinct from trogocytosis (Zimmer et al., 2003; Gaitanos et al., 2016). Under these conditions, Gulp1ΔC had no effect on ephrinB1 endocytosis, pointing to a rather specific effect of Gulp1 on trogocytosis (Fig. S2, H and I). These results show that Gulp1ΔC serves as a dominant-negative form of Gulp1 and blocks trogocytosis when enriched at EphB2/ephrinB1 clusters.

In primary cultured neurons endogenously expressing ephrinBs, reverse trogocytosis promotes rapid neurite detachment from opposing cells (Zimmer et al., 2003; Gaitanos et al., 2016). We therefore asked whether Gulp1 is also involved in this process. Cocultures of EphB2ΔC-mCherry^+^ HeLa donor cells with embryonic cortical neurons overexpressing either GFP-Gulp1ΔC, or GFP alone as a control, were live-imaged and EphB2 trans-endocytosis was quantified to evaluate the extent of reverse trogocytosis (Fig. 3, D and E; and Videos 5 and 6). We observed that GFP-Gulp1ΔC was clearly recruited to and enriched at EphB2 clusters. In the presence of GFP, EphB2 trans-endocytosis was observed in ∼50% of contact events with donor cells, whereas the presence of GFP-Gulp1ΔC largely blocked this effect. Similarly, reverse trogocytosis was inhibited when silencing endogenous Gulp1 in primary cultured neurons (Fig. 3, F–I). These results indicate that Gulp1 is required for reverse trogocytosis in physiologically relevant cell types. The PTB domain of Gulp1 is responsible for its interaction with EphB2 (Fig. S3 B) and ephrinB1 (data not shown), while the C-terminal leucine-rich region of Gulp1 may interact with effector proteins that are necessary for activating the trogocytosis process.

### Tiam2 cooperates with Gulp1 to facilitate Eph/ephrin trogocytosis

**Figure 4.**
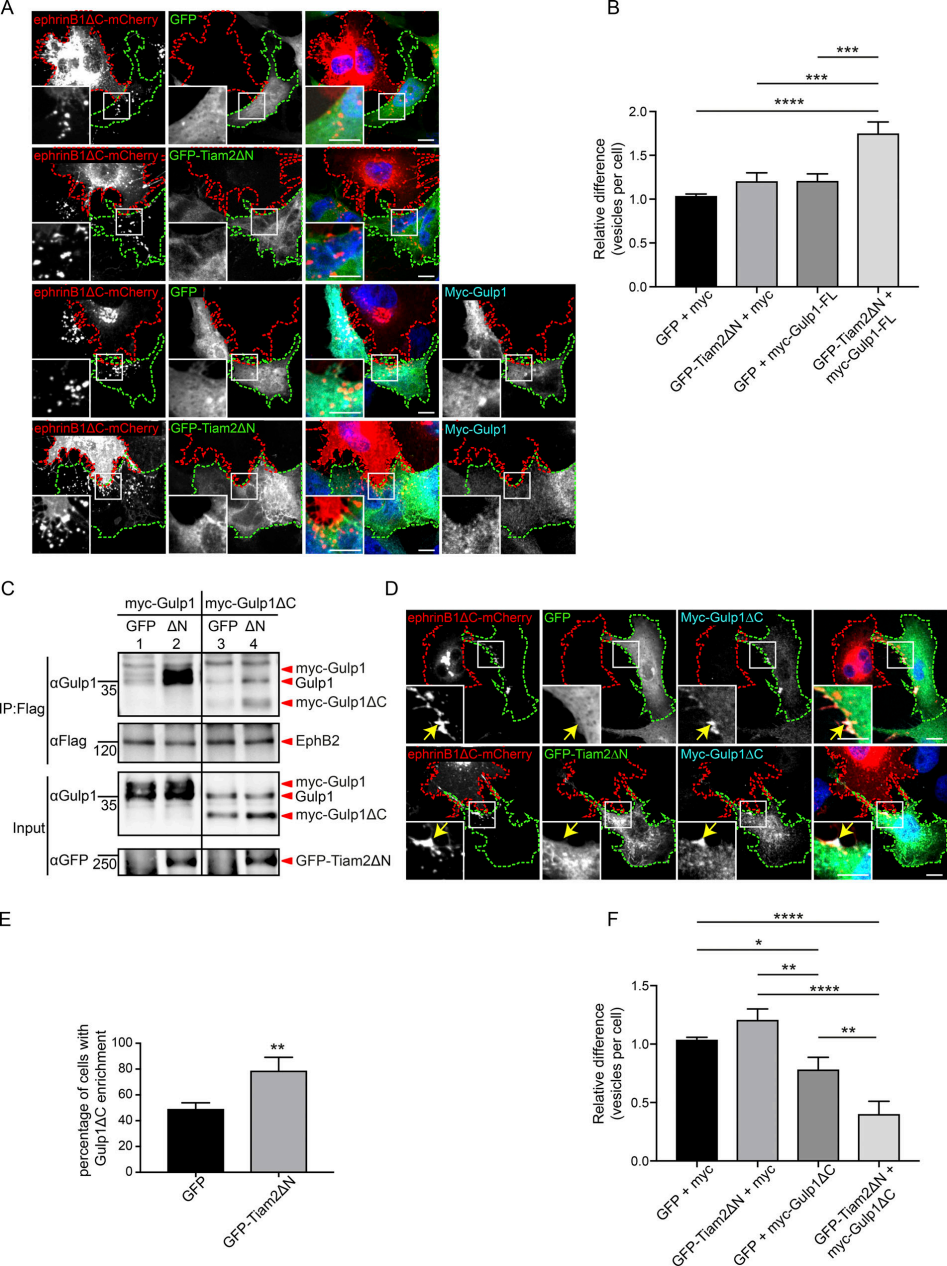
**Gulp1 cooperates with Tiam2 to facilitate EphB2/ephrinB1 trogocytosis. (A and B)** Representative images (A) and quantification (B) of coculture assays shows constitutively active Tiam2 (GFP-Tiam2ΔN) boosts the forward trogocytosis gain of function effect seen upon overexpression of Gulp1-FL. Responder cells (green dashed line) overexpressing Flag-EphB2 and myc-Gulp1 or myc (as a control), together with either GFP-Tiam2ΔN or GFP (as control), were cocultured with ephrinB1ΔC-mCherry^+^ donor cells (red dashed line). Scale bars, 10 µm. Relative values of vesicle numbers per cell shown as mean ± SEM (*n* = 3 independent experiments, 16–34 responder cells per condition per experiment). Data normalized to median GFP/myc value per experiment. ***, P < 0.001; ****, P < 0.0001; one-way ANOVA with Tukey’s multiple comparisons test. **(C)** Coimmunoprecipitation and Western blots analysis showing GFP-Tiam2ΔN (ΔN) enhances the interaction between Gulp1 and EphB2. HeLa cells were transfected with Flag-EphB2, in combination with either full-length myc-Gulp1 or myc-Gulp1ΔC, and either GFP-Tiam2ΔN or GFP. **(D and E)** Representative images (D) and quantification (E) of coculture assays showing GFP-Tiam2ΔN enhances Gulp1ΔC enrichment at ephrinB1ΔC clusters. Responder cells (green dashed lines) overexpressing Flag-EphB2 and myc-Gulp1ΔC, together with either GFP-Tiam2ΔN or GFP (as control), were cocultured with ephrinB1ΔC-mCherry^+^ donor cells (red dashed lines). Arrows indicate ephrinB1ΔC clusters. Percentage of cells with myc-Gulp1ΔC enrichment at ephrinB1ΔC clusters shown as mean ± SEM (*n* = 3 independent experiments, 17–32 responder cells per condition per experiment). **, P = 0.0099, two-tailed unpaired *t* test. **(F)** Quantification of coculture assays showing GFP-Tiam2ΔN potentiates inhibition of forward trogocytosis by Gulp1ΔC. *, P < 0.05; **, P < 0.01; ***, P < 0.001; ****, P < 0.0001; one-way ANOVA with Tukey’s multiple comparisons test.

Previously, we reported that the Rac-GEF Tiam2 is an important regulator of EphB/ephrinB trogocytosis through controlling Rac GTPase activity and thereby modulating actin polymerization (Gaitanos et al., 2016). We therefore asked whether Tiam2 and Gulp1 cooperate to regulate EphB/ephrinB trogocytosis. Overexpression of a constitutively active Tiam2 truncation (GFP-Tiam2ΔN) in EphB2^+^ responder cells led to a modest increase of forward trogocytosis (Fig. 4, A and B). A similar effect was observed when overexpressing full-length myc-Gulp1 in EphB2^+^ responder cells. Interestingly, combined overexpression of GFP-Tiam2ΔN and myc-Gulp1 in EphB2^+^ responder cells further enhanced forward trogocytosis, suggesting synergistic interactions. By using coimmunoprecipitation analysis, we found that constitutively active GFP-Tiam2ΔN, unlike the GFP control, significantly enhanced the interaction between EphB2 and Gulp1 (both endogenous and overexpressed myc-Gulp1; Fig. 4 C, compare lanes 1 and 2). These results suggest that the observed synergism between GFP-Tiam2ΔN and Gulp1 may be the result of stronger Gulp1 recruitment to EphB2 receptors in the presence of active Tiam2.

Active Tiam2 enhanced not only the interaction between EphB2 and full-length Gulp1 but also Gulp1ΔC (Fig. 4 C, compare lanes 3 and 4). Hence, in the coculture assay, a larger fraction of cells (79 ± 6%) showed enrichment of myc-Gulp1ΔC at EphB/ephrinB clusters in the presence of active GFP-Tiam2ΔN, as compared with GFP controls (49 ± 3%; Fig. 4, D and E). Consequently, inhibition of forward trogocytosis by Gulp1ΔC was more effective in the presence of active GFP-Tiam2ΔN than by Gulp1ΔC alone (Fig. 4 F). This stronger inhibition may be the result of Tiam2-mediated recruitment of Gulp1ΔC to clustered EphB2 receptors.

**Figure 5.**
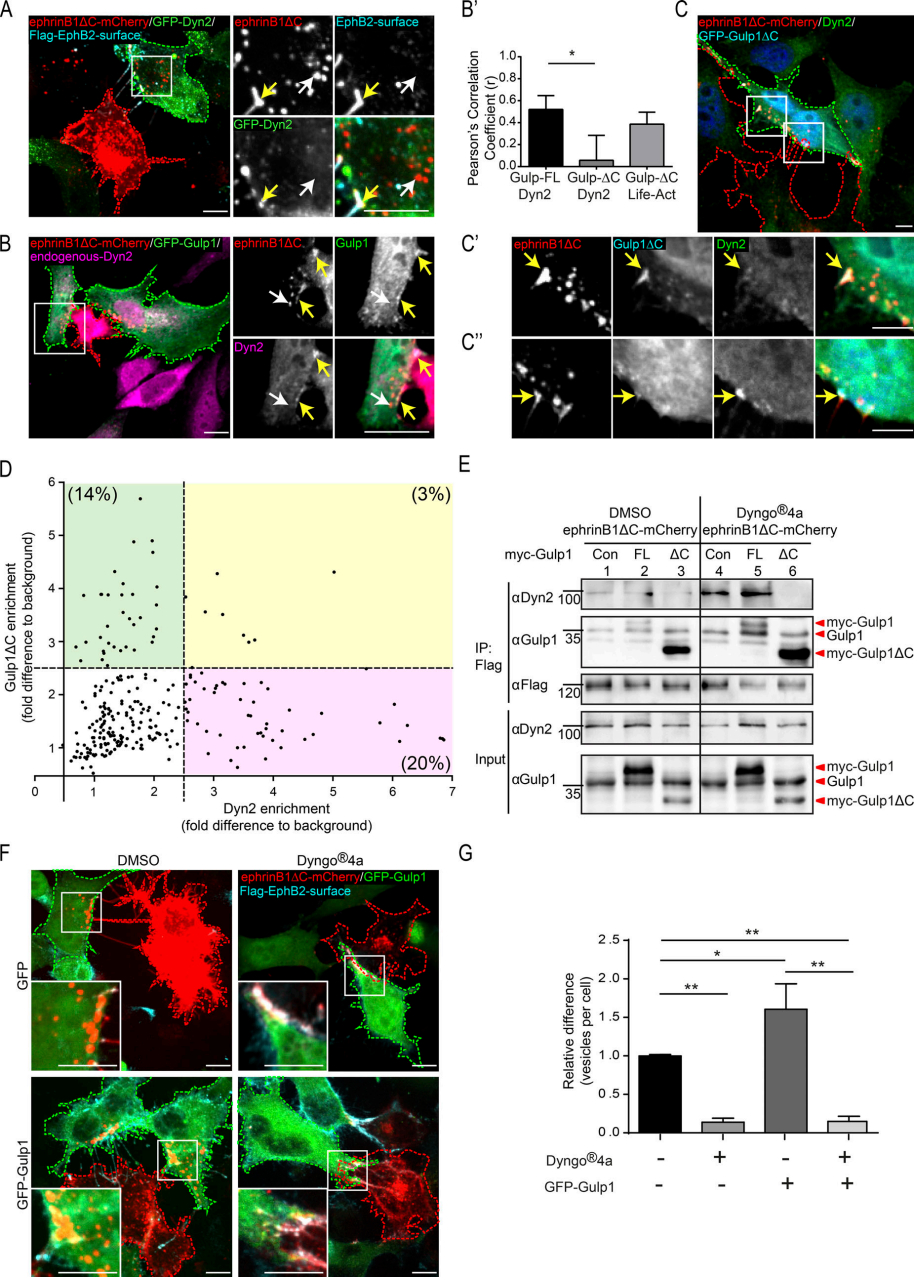
**Gulp1 regulates EphB2/ephrinB1 trogocytosis through dynamin. (A)** Representative images showing Dyn2 enriches at ephrinB1 clusters during forward trogocytosis in HeLa cells. Responder cells (green dashed line) overexpressing Flag-EphB2 with WT Dyn2 (GFP-Dyn2) were cocultured with ephrinB1ΔC-mCherry^+^ donor cells (red dashed line). **(B)** Representative images showing endogenous Dyn2 and GFP-Gulp1 colocalize at ephrinB1 clusters during forward trogocytosis in HeLa cells. Responder cells (green dashed line) overexpressing Flag-EphB2 with GFP-Gulp1 were cocultured with ephrinB1ΔC-mCherry^+^ donor cells (red dashed line), and endogenous Dyn2 was immunostained. In A and B, white arrows indicate internalized vesicles and yellow arrows indicate surface clusters. **(B′)** Pearson’s correlation coefficient analysis for the colocalization between endogenous Dyn2 and GFP-Gulp1-FL or GFP-Gulp1ΔC, and between Life-Act and GFP-Gulp1ΔC at ephrinB1ΔC clusters for experiments described in B, C, and Fig. S3 D, respectively. Maximum fluorescence intensities of Dyn2, Life-Act, and GFP-Gulp1 or GFP-Gulp1ΔC within each ephrinB1ΔC cluster regions of interest were measured and normalized to their respective cell background signal. *n* = 3 independent experiments, 4–12 cells per experiment. *, P < 0.05, one-way ANOVA with Tukey’s multiple comparisons test. **(C)** Representative images showing Gulp1ΔC disrupts endogenous Dyn2 recruitment to ephrinB1 clusters. Responder cells (green dashed line) overexpressing Flag-EphB2 with GFP-Gulp1ΔC were cocultured with ephrinB1ΔC-mCherry^+^ donor cells (red dashed line) and endogenous Dyn2 was immunostained. Enlarged insets show reduced Dyn2 signal at ephrinB1 clusters with Gulp1ΔC colocalization (C′) compared with clusters without Gulp1ΔC colocalization (C″). Arrows indicate ephrinB1ΔC clusters. **(D)** Quantification of Dyn2 and Gulp1ΔC enrichment at ephrinB1 clusters. Maximal fluorescence intensities of Dyn2 and GFP-Gulp1ΔC within each ephrinB1 cluster regions of interest were measured and normalized to background (value from a region in the same responder cell without ephrinB1 clusters or vesicles). Each point represents an independent cluster. Threshold to indicate enrichment was set to 2.5 times that of background. *n* = 4 independent experiments, 4–10 cells per experiment. **(E)** Coimmunoprecipitation and Western blot analysis showing the formation of EphB2/Gulp1/Dyn2 complexes during forward trogocytosis, and Gulp1ΔC blocking recruitment of Dyn2 to EphB2 complexes. Responder HeLa cells expressing Flag-EphB2, in combination with myc (as a control [Con]), full-length myc-Gulp1 (FL), or myc-Gulp1ΔC (ΔC), were treated with either DMSO or Dyngo-4a before being cocultured with ephrinB1ΔC-mCherry^+^ donor cells. **(F and G)** Representative images (F) and quantification (G) showing inhibition of dynamin activity blocks forward trogocytosis in HeLa cells. Quantification results shown as mean ± SEM (*n* = 3 independent experiments, 9–20 responder cells per condition per experiment, normalized to median GFP^+^ DMSO value per experiment). *, P < 0.05; **, P < 0.01; one-way ANOVA with Tukey’s multiple comparisons test. Scale bars in A–C and F, 10 µm.

Support for cooperation between Tiam2 and Gulp1 was also revealed using dominant-negative Tiam2 (Tiam2ΔNDN), a point mutant of Tiam2 that lacks RacGEF activity (Gaitanos et al., 2016). In the presence of Tiam2ΔNDN, the fraction of cells with myc-Gulp1ΔC enrichment at EphB/ephrinB clusters after coculture with ephrinB1ΔC-mCherry^+^ donor cells dropped to <20% (Fig. S3, A and B), illustrating that the RacGEF activity of Tiam2 contributes to the recruitment of Gulp1ΔC to EphB2 clusters. Consequently, the effect of coexpressing Gulp1ΔC and Tiam2ΔNDN did not further reduce forward trogocytosis compared with Tiam2ΔNDN alone (Fig. S3 C). Furthermore, when we co-overexpressed LifeAct-mCherry in GFP-Gulp1ΔC and EphB2^+^ donor cells, we still consistently observed efficient actin polymerization at EphB2/ephrinB1 clusters upon cell–cell contact, despite enrichment of dominant-negative Gulp1ΔC at the same clusters (Fig. S3, D and E; and Fig. 5 B′), suggesting that Gulp1 is not involved in modulating Rac GTPase and actin polymerization during trogocytosis. Together, these results confirm that Tiam2 cooperates with Gulp1 to promote forward trogocytosis, likely through stabilizing the recruitment of Gulp1 to EphB2/ephrinB1 clusters.

### Dynamin is necessary for Gulp1 to regulate Eph/ephrin trogocytosis

Gulp1 was thought to mediate phagocytosis by regulating dynamin activity (Yu et al., 2006). Because dynamin has also been implicated in Eph/ephrin signaling (Cowan and Henkemeyer, 2001; Parker et al., 2004; Yu et al., 2006; Kida et al., 2007; Evergren et al., 2018), we asked whether Gulp1’s regulation of Eph/ephrin trogocytosis acts via dynamin. First, we investigated whether dynamin2 (Dyn2) is recruited to EphB2/ephrinB1 clusters in our cell–cell stimulation assay. Overexpressed GFP-Dyn2 in EphB2^+^ HeLa responder cells was clearly recruited to ephrinB1ΔC surface clusters but not to internalized ephrinB1ΔC^+^ vesicles (Fig. 5 A). Similar results were obtained for endogenous Dyn2 in U251 cells endogenously expressing EphB2 (Fig. S4 A). Conversely, endogenous Dyn2 was recruited to surface EphB2ΔC clusters in SKN cells endogenously expressing ephrinBs (Fig. S4 B).

Next, we investigated the interaction of Dyn2 and Gulp1 during EphB2/ephrinB1 trogocytosis. Pearson’s correlation coefficient analysis showed significant correlation between endogenous Dyn2 signals and transfected GFP-Gulp1 at ephrinB1ΔC clusters (Fig. 5, B and B′). Moreover, endogenous Dyn2 coimmunoprecipitated in complex with EphB2 and Gulp1 when EphB2-expressing cells were cocultured with ephrinB1ΔC^+^ donor cells (Fig. 1 D). Likewise, endogenous dynamin coimmunoprecipitated in complex with endogenous ephrinBs and Gulp1 when ephrinB-expressing cells were cocultured with EphB2ΔC^+^ donor cells (Fig. S1 F).

Next, we analyzed the localization of Dyn2 in cells when trogocytosis was inhibited by Gulp1ΔC. As described above (Fig. 3 C), colocalization of Gulp1ΔC with surface EphB2/ephrinB1 clusters was observed in about half of the transfected cells. Whenever there was efficient Gulp1ΔC enrichment at ephrinB1ΔC clusters, colocalization of endogenous Dyn2 was low (Fig. 5, C, C′, and D). Conversely, whenever Dyn2 colocalization was prominent, there was little Gulp1ΔC detectable at ephrinB1ΔC clusters, even within cells that showed Gulp1ΔC enrichment at some contact sites (Fig. 5, C″ and D). Only around 3% of ephrinB1ΔC clusters had significant enrichment of both Gulp1ΔC and Dyn2 at the same time (Fig. 5 D). Pearson’s correlation coefficient analysis confirmed lack of colocalization between Gulp1ΔC and dynamin at ephrinB1ΔC clusters (Fig. 5 B′). Further quantification showed that ephrinB1ΔC clusters with dynamin enrichment were closer to the cell surface than ephrinB1ΔC vesicles without dynamin enrichment (Fig. S4 C).

Gulp1ΔC interference of dynamin binding to EphB2 could also be observed in coimmunoprecipitation experiments. When Gulp1ΔC was overexpressed during EphB2/ephrinB1 trogocytosis, Dyn2 no longer coimmunoprecipitated with EphB2 (Fig. 5 E, compare lanes 2 and 3). This effect was more obvious when the EphB2/Gulp1/Dyn2 complex was stabilized in the presence of the specific dynamin inhibitor Dyngo-4a (Fig. 5 E, compare lanes 5 and 6).

Next, we asked whether Dyn2 was required for EphB2/ephrinB1 trogocytosis in our paradigm. When dynamin activity was inhibited by Dyngo-4a, forward trogocytosis was largely blocked as compared with vehicle treatment (13.9 ± 5% of control; Fig. 5, F and G). Similar results were obtained with overexpression of dominant-negative GFP-Dyn2 (data not shown). Importantly, dynamin inhibition did not interfere with EphB2/ephrinB1 clustering. Moreover, inhibition of dynamin activity by Dyngo-4a also blocked the gain-of-function effect of overexpressed full-length Gulp1, suggesting that dynamin is essential for Gulp1 function during forward trogocytosis. Similar experiments in ephrinB-expressing SKN cells showed that inhibition of dynamin activity blocked reverse trogocytosis (Fig. S4, D and E). Overall, these results show that Gulp1 acts by recruiting dynamin to promote Eph/ephrin trogocytosis, a process directly dependent on dynamin function.

### Gulp1 is required for EphB2/ephrinB1-mediated cell disengagement

**Figure 6.**
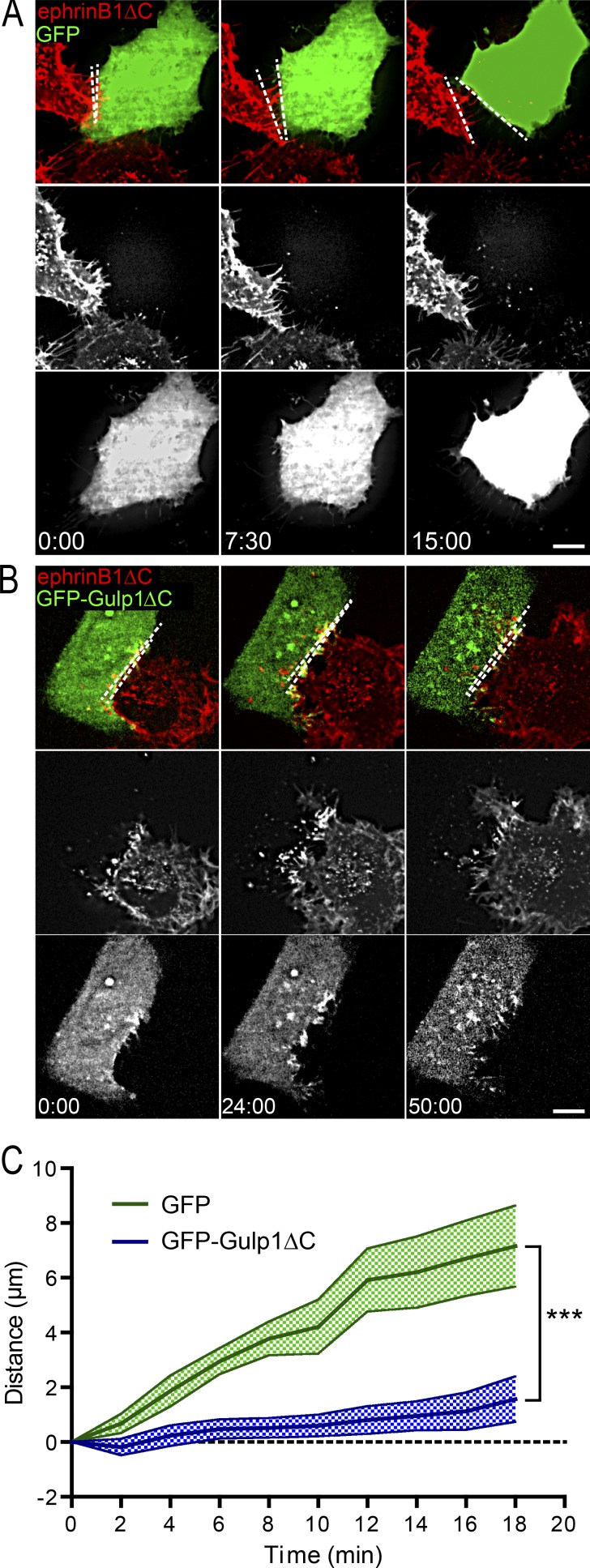
**Gulp1 regulates the EphB2/ephrinB1-mediated cell disengagement response. (A–C)** Time-lapse images (A and B) and quantification (C) showing expression of dominant-negative Gulp1ΔC inhibits EphB2/ephrinB1-mediated cell disengagement. HeLa cells expressing EphB2 and either GFP or GFP-Gulp1ΔC were cocultured with donor cells expressing ephrinB1ΔC-mCherry. Maximum projection of deconvolved images is shown. Scale bars, 10 µm. Elapsed time shown as min:s. Dashed lines indicate the distance between two contacting cells. Measured distance between donor and responder cells over time for each condition shown as mean ± SEM *n* = 20 (GFP-Gulp1ΔC) and 13 (GFP) donor–responder pairs from three experiments. ***, P < 0.001; two-way ANOVA.

EphB2/ephrinB1 trogocytosis is required for rapid cell detachment and for efficient cell repulsion to occur (Zimmer et al., 2003; Gaitanos et al., 2016). Having demonstrated that Gulp1 is an essential player in EphB2/ephrinB1 trogocytosis, we investigated how Gulp1 influences the cell detachment response. We performed live imaging on EphB2^+^ HeLa cells also overexpressing either GFP or GFP-Gulp1ΔC cocultured with ephrinB1ΔC-mCherry^+^ HeLa donor cells and measured the distance between responder and donor cells over time after an initial contact. In the GFP control condition, after cell–cell contact and forward trogocytosis, responder cells readily disengaged from the ephrinB1^+^ donor cells (Fig. 6, A and C; and Video 7). In contrast, GFP-Gulp1ΔC–expressing EphB2^+^ responder cells remained in direct contact with ephrinB1^+^ cells (Fig. 6, B and C; and Video 8). These data illustrate that Gulp1-mediated forward trogocytosis is required for Eph/ephrin-driven cell contact–mediated disengagement.

### Gulp1 is involved in the movement of vegetal rotation in *Xenopus* gastrula

**Figure 7.**
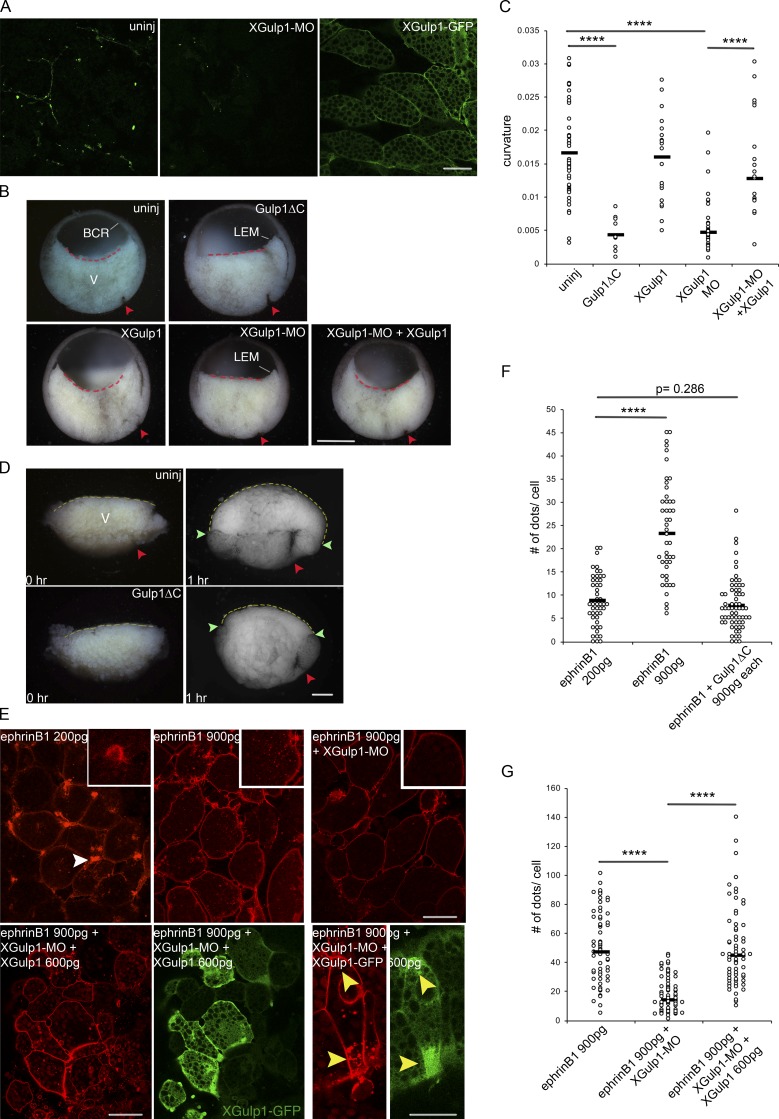
**Role of Gulp1 in *Xenopus* gastrulation. (A)** Expression of XGulp1 protein in vegetal cells. Anti-Gulp1 antibody staining without or with knockdown by XGulp1-MO (left and middle panels) and localization of XGulp1-GFP (300 pg, the right panel). Scale bar, 50 µm. **(B)** Mid-sagittal fractures of *Xenopus* early gastrulae. The central endodermal blastocoel floor is curved in uninjected, XGulp1-GFP–injected, or XGulp1-MO/XGulp1-GFP–coinjected embryos, but straight in Gulp1ΔC expressing or XGulp1-MO gastrulae (dashed lines; XGulp1-MO, 30 ng; XGulp1-GFP, 900 pg). Red arrowheads, dorsal blastopore; BCR, blastocoel roof; LEM, leading edge mesendoderm that begins to advance on the BCR; V, endoderm of vegetal cell mass. Scale bar, 500 µm. **(C)** Curvature of blastocoel floor. Embryos from three independent experiments were measured by the Kappa plugin in ImageJ, and results were pooled for each treatment. **(D)** Explants of mid-sagittal slices of vegetal cell mass fixed immediately after excision (0 hr) and after onset of vegetal rotation (1 hr) uninjected (top) or after Gulp1ΔC mRNA injection (bottom). Yellow dashed lines, endodermal blastocoel floor surface. Average lengths of dashed lines from three independent experiments: 2,381.0 ± 254.5 µm for eight uninjected explants; 1,862.0 ± 285.5 µm for nine Gulp1ΔC mRNA injected explants. Red arrowheads, dorsal blastopore; green arrowheads, border between surface of embryo and blastocoel floor. Scale bar, 200 µm. **(E)** Cells in vegetal slice explants after injection of ephrinB1-mCherry mRNA alone or together with XGulp1-MO or XGulp1-MO and XGulp1-GFP. Amount of mRNA injected per blastomere is indicated. Explants from three sets of experiments were examined by confocal microscopy. Additional explants analyzed by conventional fluorescence microscopy gave the same results (not shown). White arrowheads point to retracting protrusions, yellow arrowheads to cell regions simultaneously enriched in vesicles and XGulp1-GFP. Scale bars, 50 µm (left) and 30 µm (right). **(F and G)** Number of intracellular dots per cell. Asterisks indicate significance levels: ****, P < 0.0001; not significant, P = 0.286; two-tailed Student’s *t* test.

Next, we asked if Gulp1 plays a role in ephrinB1 trogocytosis-dependent cell movement in vivo. During the vegetal rotation movement in the *Xenopus* gastrula, endoderm cells rearrange by migrating across each other (Winklbauer and Schürfeld, 1999). In the process, ephrinB1-dependent trogocytosis and macropinocytosis are required to allow for the detachment and retraction of the trailing edges of the cells (Wen and Winklbauer, 2017). Since *Xenopus* Gulp1 (XGulp1) expression peaks during gastrula stages (Session et al., 2016), and XGulp1 protein is detected in vegetal cells (Fig. 7 A), we asked whether it modulates the extent of vegetal rotation. We found that injection of mRNA encoding mouse dominant-negative Gulp1ΔC at the four-cell stage indeed affected gastrulation (Fig. 7 B). Vegetal rotation normally expands the blastocoel floor, and within the confines of the embryo, the floor becomes concave as mesoderm and endoderm cells accumulate at the blastocoel roof (Fig. 7 B). Like ephrinB1 knockdown (Wen and Winklbauer, 2017), Gulp1ΔC completely abolished this shape change of the endoderm and left the blastocoel floor flat (Fig. 7, B and C), indicating interference with vegetal rotation (Wen and Winklbauer, 2017). Likewise, knockdown of endogenous XGulp1 with morpholino antisense oligonucleotide directed against the 5′ UTR of XGulp1 mRNA (XGulp1-MO) diminished XGulp1 protein expression in vegetal cells (Fig. 7 A) and inhibited vegetal rotation (Fig. 7, B and C). Overexpressed XGulp1-GFP localized to cell membranes and also cytoplasmically between the yolk platelets (Fig. 7 A) but did not affect gastrulation, and coinjection of MO-resistant XGulp1-GFP mRNA with XGulp1-MO rescued vegetal rotation (Fig. 7, B and C; and Fig. S5 A).

When slices of the vegetal endoderm are explanted at the onset of vegetal rotation, uninjected explants bulge upward in the absence of the blastocoel roof while the mesodermal region is moved downward by the migration and consequent rearrangement of the vegetal cells (Fig. 7 D) as previously shown (Winklbauer and Schürfeld, 1999; Wen and Winklbauer, 2017). Gulp1ΔC-injected explants, by contrast, rounded up but did not expand their blastocoel floor (Fig. 7 D), consistent with the absence of vegetal rotation. As seen at later stages, mesoderm internalization is also attenuated, whereas blastopore formation, mesendoderm translocation across the ectoderm, or mesoderm–ectoderm boundary formation was not affected (leading edge mesendoderm in Fig. 7 B, and data not shown).

Next, we asked whether Gulp1 is involved in the ephrinB1-dependent uptake of cell membrane. In vegetal endoderm cells, knocking down ephrinB1 diminishes the membrane internalization and cell-on-cell migration required for the vegetal rotation movement, whereas ephrinB1 overexpression strongly increases membrane uptake and leads to cell detachment and rounding, which in turn also impedes cell migration and vegetal rotation (Wen and Winklbauer, 2017). At a low level of expression, ephrinB1-mCherry has no apparent effects on cell behavior. It localizes to the cell membranes, in particular at flat, retracting protrusions of cell tails, and to puncta within cells that have previously been identified as single- or double-membrane vesicles (Wen and Winklbauer, 2017; Fig. 7 E). At high expression levels, cells began to round up and puncta became much more numerous (Fig. 7, E and G), confirming our previous findings. The ephrinB1-induced increase in puncta was reversed by coinjection of XGulp1-MO (Fig. 7, E and G) or coexpression of Gulp1ΔC (Fig. 7 F and Fig. S5 B), demonstrating a requirement for Gulp1 in ephrinB1-dependent membrane uptake in *Xenopus* gastrulation. Cells also appeared to be more tightly attached to each other and less protrusive, as previously seen in ephrinB1-depleted tissue (Wen and Winklbauer, 2017). Triple injection of ephrinB1-mCherry mRNA, XGulp1-MO, and XGulp1-GFP reversed the effect of XGulp1 knockdown and increased vesicles number again (Fig. 7, E and G). These results provide evidence that XGulp1 is required for ephrinB1-dependent membrane uptake, a process including trogocytosis in vivo, which in turn regulates Eph/ephrin-mediated cell repulsion and cell rearrangement during vegetal rotation in the *Xenopus* gastrula.

## Discussion

Here we report that the engulfment adaptor protein Gulp1 promotes EphB/ephrinB trogocytosis and cell rearrangements of cultured cells both in vitro and in vivo. These, together with previous, results (Gaitanos et al., 2016) suggest that EphB/ephrinB trogocytosis uses phagocytosis-like intracellular pathways to achieve efficient membrane scission and engulfment. These observations diverge from previous reports of other types of trogocytosis that have highlighted distinct mechanisms (Abdu et al., 2016; Somlata et al., 2017). Our results therefore suggest that different forms of trogocytosis exist: those that resemble phagocytosis, and others that are rather distinct. Previously, Gulp1 function has been shown in different forms of phagocytosis, including phagocytic astrocytes in the ischemic brain (Koizumi et al., 2018) and phagocytic glial precursors in the developing peripheral nervous system (Sullivan et al., 2014). Interestingly, in *C. elegans*, Gulp1 has been found to be required for phagocytosis of apoptotic cell corpses (Liu and Hengartner, 1998), but not for trogocytosis of PGC protrusions by endodermal cells (Abdu et al., 2016).

**Figure 8.**
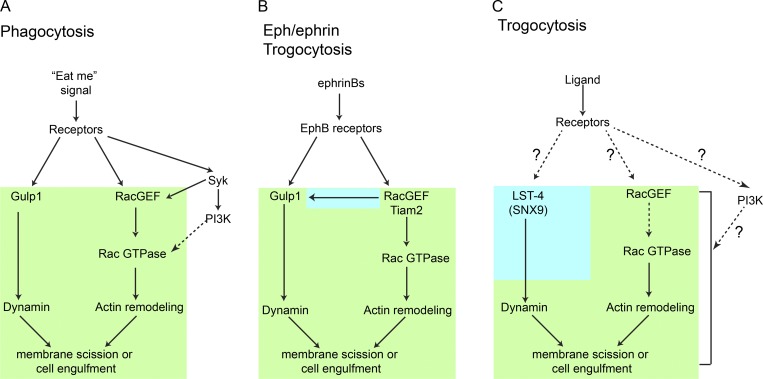
**Models comparing Eph/ephrin trogocytosis to apoptotic phagocytosis and classical trogocytosis.** Schematic representation of molecular pathways mediating phagocytosis (A), Eph/ephrin-mediated trogocytosis (B), and immune and germ cell trogocytosis (C) showing that Eph/ephrin-mediated trogocytosis shares both similarities (highlighted in green) and distinct properties (blue) with both processes. In all three instances, a Rac-GEF (Tiam2 in the case of Eph/ephrin trogocytosis) activates Rac GTPase to mediate the actin polymerization required for membrane rearrangement essential for internalizing large structures. Furthermore, all three pathways recruit dynamin, required for membrane scission and thereby allowing for internalization. However, in the cases of phagocytosis and Eph/ephrin trogocytosis, the engulfment protein Gulp1 is essential for dynamin recruitment, while it is not required in a classical trogocytosis setting. Moreover, in the case of Eph/ephrin trogocytosis, the stable Gulp1/EphB2 complex requires an active GEF (again using Tiam2) before dynamin recruitment. Dotted lines indicate indirect evidence.

Our dissection of Gulp1 function during EphB/ephrinB-mediated trogocytosis revealed some interesting similarities to the apoptotic phagocytic pathways originally revealed in *C. elegans*. Genetic analysis suggests that in *C. elegans*, dynamin1 acts in the same genetic pathway as the ced-6/Gulp1 and ced-1/scavenger receptors (Yu et al., 2006). Ced-6/Gulp1 either directly interacts with dynamin1, or regulates its activity via an unidentified ced-6/Gulp1-interacting protein. The phenotype displayed by *C. elegans* dynamin1 mutants was partially rescued with human Dyn2, suggesting that the function of dynamins in the removal of apoptotic cells may be conserved from worms to mammals (Yu et al., 2006). ced-5/Dock180, ced-12/ELMO, and ced-10/Rac1 were not required for the recruitment of dynamin1 to the site of engulfment (Yu et al., 2006). We found that Gulp1 mediates dynamin’s function, as dynamin is no longer enriched at EphB/ephrinB clusters, nor forms a complex with activated EphB2 receptors when dominant-negative Gulp1 is present. Actin is still efficiently polymerized in this scenario, suggesting that Rac1 can be activated normally when Gulp1 is not functional (Fig. 8). A key activator of Rac1 during EphB/ephrinB trogocytosis is the Rac-GEF Tiam2, which activates Rac GTPase activity to mediate actin polymerization (Gaitanos et al., 2016). In this study, we found that Tiam2 regulates the formation of Gulp1/EphB2 complex in its GEF activity-dependent manner. Whereas constitutively active Tiam2 enhances the recruitment of Gulp1 to Eph/ephrin clusters, dominant-negative Tiam2 leads to reduced binding of Gulp1 to Eph/ephrin clusters. This suggests that Gulp1 is in fact a downstream effector of the Rac GTPase in Eph/ephrin trogocytosis.

Gulp1 also plays roles in EphB/ephrinB-mediated trogocytosis in the intact *Xenopus* gastrula. Previously, gastrulating endodermal cells have been shown to move by amoeboid shape changes, including retraction of their trailing edge, which involves ephrinB1-dependent macropinocytosis and trogocytosis (Wen and Winklbauer, 2017). Here, by using Gulp1 loss-of-function approaches, we have shown that it is required for endoderm migration and ephrinB1-dependent membrane uptake. In future work, it would be interesting to investigate whether Gulp1 plays a role in other Eph/ephrin-mediated trogocytosis events, such as developmental axon pruning (Xu and Henkemeyer, 2009) and synapse remodeling by astrocytes during learning and memory (Koeppen et al., 2018). Consistent with the latter hypothesis, Gulp1 expression in astrocytes is comparatively high (Zhang et al., 2014).

Spatio-temporal control of the resolution of cell–cell contact is important to ensure efficient cell repulsion. In the Eph/ephrin system, signaling and response require the formation of higher-order Eph/ephrin clusters (Schaupp et al., 2014). If scission occurs too early, the loss of contact may result in no retraction signal being activated. Alternatively, a delay in scission could result in formation of overly large clusters that may not be able to be internalized and cleared. Gulp1’s position between the activation of Rac via Tiam2 that leads to the formation of the actin-rich phagocytic cup, and the recruitment of dynamin required for vesicle scission and relief from the tight cell–cell contact, places it at the breaking point between signal amplification and cell response.

## Materials and methods

### Plasmids

Expression constructs encoding murine full-length or C-terminally truncated EphB2 (untagged or N-terminal Flag-tagged) or ephrinB1 (N-terminal HA-tagged), and LifeAct-mCherry-N1 have been described previously (Zimmer et al., 2003; Gaitanos et al., 2016). Rat WT pEGFP-Dyn2 were gifts from S. Schmid (Department of Cell Biology, University of Texas Southwestern, Dallas, TX) plasmid 34686; Addgene). Mouse constitutively active GFP-Tiam2ΔN and dominant-negative GFP-Tiam2ΔNDN were gifts from A. Malliri (Cancer Research UK, Manchester, UK).

Full-length cDNA encoding Gulp1 was cloned from cDNA of NIH/3T3 cells and subcloned into pCMV-myc or pEGFP-C3 vectors. Truncation fragments of Gulp1 were amplified by PCR using appropriate primers from full-length myc-Gulp1 and subcloned into XhoI/EcoRI sites of pEGFP vectors. For Gulp1 expression in *Xenopus* embryos, the vectors containing EGFP-tagged full length or a C-terminally truncated form (ΔC) of mouse Gulp1 were digested with both XhoI and HpaI. The coding sequence and poly-A tail of the full length and ΔC from EGFP-Gulp1 were then subcloned into the XhoI/SnaBI sites of the pCS2^+^ plasmids. The fidelity of all plasmid DNA constructs was verified by sequencing analysis. The transformed pCS2^+^-GFP-Gulp1 and pCS2^+^-GFP-Gulp1ΔC were linearized with BssHII and used for in vitro transcription with SP6 polymerase. The XGulp1-GFP DNA sequence was de novo synthesized by Eurofins Genomics and subcloned into the Stul site of pCS2^+^ using the SLIC (sequence- and ligation-independent cloning) method (Li and Elledge, 2012), and the sequence was validated. pCS2^+^-XGulp1-GFP was linearized with NotI and transcribed with SP6.

### Antibodies and reagents

Antibodies used were as follows: anti-Gulp1 (HPA020587; rabbit polyclonal; Sigma-Aldrich), anti-GFP (JL-8; mouse monoclonal; Clontech), anti-C-Myc (SC-40; mouse monoclonal; Santa Cruz), anti-β-tubulin (480011; mouse monoclonal; Thermo Fisher Scientific), anti-Dyn2 (ab3457; rabbit polyclonal; Abcam), M2 anti-FLAG (F3165; mouse monoclonal; Sigma-Aldrich), anti-FLAG (F7425; rabbit polyclonal; Sigma-Aldrich), and anti-HA (ab18181; mouse monoclonal; Abcam). Secondary antibodies purchased from Jackson ImmunoResearch Laboratories, including HRP-coupled secondary antibodies, were used for Western blots, and fluorescently labeled secondary antibodies were used for immunostaining. For STED imaging, Abberior Star 580 and Abberior Star Red secondary antibodies were purchased from Abberior Instruments.

For soluble ligand stimulation, Human IgG Fc fragment (Jackson ImmunoResearch Laboratories) or mouse ephrinB1-Fc fusion protein (R&D Systems) was incubated with goat anti-human Fc at a ratio of 5:1 for 30 min (Zimmer et al., 2003). Cells were incubated with the clustered proteins at a final concentration of 2 µg/ml for 30 min at 37°C.

Dynamin inhibitor Dyngo-4a (ab120689; Abcam) was diluted in DMSO.

### Cell culture, transfection, and inhibitor treatment

HeLa (human epithelial adenocarcinoma, ATCC CCL-2) cells and 293 (HEK-293; ATCC CRL-1573) cells were cultured in DMEM (Gibco) containing 10% fetal bovine serum (Gibco) and 1% penicillin/streptomycin (Thermo Fisher Scientific) and transfected using Lipofectamine 2000 (Invitrogen) according to the manufacturers’ instructions.

U251 cells (09063001-1VL; Sigma-Aldrich) were cultured in MEM (Gibco) containing 10% fetal bovine serum (Gibco), 2 mM glutamine (Thermo Fisher Scientific), 1% nonessential amino acids (Thermo Fisher Scientific), and 1 mM sodium pyruvate (Thermo Fisher Scientific).

SKN cells were cultured in Opti-MEM with Glutamax (Gibco) supplemented with 10% fetal bovine serum (Gibco) and 1% penicillin/streptomycin (Thermo Fisher Scientific).

Primary cortical neurons were dissected from embryonic day 15.5 mouse embryos, plated onto cell culture dishes coated with 1 mg/ml poly-D-lysine (Sigma-Aldrich) and 5 µg/ml laminin (Gibco), and cultured in Neurobasal medium (Gibco) supplemented with 2% B27 supplement (Gibco), 10 mM glutamine (Thermo Fisher Scientific), and 1% penicillin/streptomycin (Thermo Fisher Scientific), and incubated at 37°C and 5% CO_2_. Primary cultured cortical neurons were transfected using the Calcium phosphate transfection kit (Invitrogen) according to the manufacturer’s protocol at 2 d in vitro.

For Gulp1 knockdown in HeLa cells, stealth siRNA oligos (1299001; HSS122222; sequence: 5′-CCA​GUC​UUC​GAU​GCC​UAC​UCG​CAA​U-3′; Invitrogen) were reverse-transfected using Lipofectamine RNAiMax (Invitrogen) transfection reagent according to the manufacturer’s instructions. For Gulp1 knockdown in primary cultured cortical neurons, stealth siRNA oligos (1299001; HSS182059; sequence: 5′-CAU​AUG​CAA​AGA​UUC​UGA​GUC​AAA​U-3′; Invitrogen) were transfected using Lipofectamine RNAiMax (Invitrogen) transfection reagent according to the manufacturer’s instructions at 3 d in vitro.

For dynamin inhibition, responder HeLa cells or SKN cells were treated with 15 µM dynamin inhibitor Dyngo-4a for 60 min before the cell–cell stimulation assay, and then cocultured with donor cells for 60 min in the presence of Dyngo-4a.

### Coimmunoprecipitation

HeLa cells, U251 cells, or SKN cells treated as indicated were washed twice with PBS and lysed in immunoprecipitation buffer (25 mM ​Hepes, pH7.4, 150 mM ​NaCl, and 1% Triton X-100) by sonication. Cell lysates were cleared by centrifugation and then incubated with either anti-FLAG M2-Agarose resin (Sigma-Aldrich) or protein G sepharose 4 fast flow beads (GE Healthcare) supplemented with indicated antibodies for at least 3 h at 4°C, washed four times with lysis buffer, and then analyzed by Western blot.

### Cell–cell stimulation (trogocytosis) and cell disengagement assays

For the cell–cell stimulation (trogocytosis) assays, donor HeLa cells were harvested with 0.02% EDTA in PBS without magnesium and calcium (Sigma-Aldrich) and cocultured with responder cells for 60 min at 37°C before fixation.

For the cell disengagement assay in HeLa cells, responder EphB2^+^ cells coexpressing GFP or GFP-Gulp1ΔC were cocultured with ephrinB1ΔC-mCherry^+^ HeLa donor cells and subjected to live image acquisition. Four confocal planes were taken at each position at 5-min intervals for a total duration of 2 h. Images were analyzed using MetaMorph (Molecular Devices).

For the trogocytosis assay in primary cortical neurons, neurons expressing GFP or GFP-Gulp1ΔC were cocultured with EphB2ΔC-mCherry expressing HeLa donor cells and subjected to live image acquisition. Four confocal planes were taken at each position at 1.5-min intervals. Images were analyzed using MetaMorph. Contact points between GFP-positive neurons and EphB2ΔC-mCherry^+^ HeLa cells were tracked and scored for internalization.

### Immunocytochemistry

Cells were fixed with prewarmed 4% paraformaldehyde and 8% sucrose in Dulbeccos’ PBS for 20 min at RT, rinsed twice with ice-cold PBS, and then incubated with ice-cold 50 mM ammonium chloride in PBS for 10 min and rinsed again. For surface labeling of Ephs or ephrins, cells were not permeabilized. Blocking was performed for 30 min at RT with 3% BSA in PBS, followed by incubation with the primary antibodies (anti-Flag for EphB2, and anti-HA for ephrinB1) in blocking solution for 2 h at RT. For further total labeling, cells were permeabilized with 0.1% Triton X-100 for 5 min, and incubated with blocking solution for 1 h at RT, followed by incubation with the primary antibodies in blocking solution for 2 h at RT. After washing with PBS, secondary antibodies diluted 1:500 in blocking solution were applied for 1 h at RT. After washing, coverslips were mounted using the ProLong antifade kit (Invitrogen).

### Live and fixed cell imaging

Images were collected on an Axio Observer Z1 inverted microscope (Zeiss) equipped with a CSU-X1 spinning disc confocal unit (Yokogawa Electric) controlled by VisiView software (Visitron Systems) and a CoolSnapHQ2 CCD camera (Photometrics). For live imaging, a temperature-controlled CO_2_ incubation chamber (Pecon) was used at 37°C and 5% CO_2_, and cells were imaged in normal growth media. Excitation was provided by lasers of 405, 488, 561, or 640 nm wavelength (Visitron Systems). HeLa cells on coverslips were imaged with a Plan-Achromat 40× 1.4 NA oil immersion objective or a 63× 1.4 NA oil-immersion objective (Zeiss). Z-stacks of eight planes every 0.75-µm step size were acquired. Maximum projections of fixed images were performed using Fiji software, and vesicles were determined by counting blinded images. Distances between cells in cell repulsion assays were measured in MetaMorph. For visualization purposes, all images are presented after intensity adjustment using Fiji or Photoshop (Adobe Systems). All adjustments within an experiment were performed equally.

### STED imaging and processing

For STED imaging, untagged EphB2/GFP-Gulp1ΔC^+^ cells were cocultured with HA-ephrinB1ΔC-mCherry^+^ donor cells, fixed, permeabilized, and blocked as described above. Cells were then probed with anti-GFP and anti-HA, washed and probed for secondary antibodies, and mounted on slides using ProLong antifade. Images were acquired using a STEDYCON STED unit (Abberior Instruments) mounted on a Zeiss Axio Imager Z2 upright microscope using a 100× oil immersion objective, NA = 1.46 (Zeiss). Pulsed excitation laser lines of 594 and 640 nm and a STED beam at 775 nm were used. Images were acquired at 20-nm x-y pixel size with two avalanche photodiode detectors. Z-steps of eight planes were acquired every 250 nm. Images were processed as above.

### *Xenopus* embryos, microinjections, embryo fixation, and explants

*Xenopus* embryos were fertilized in vitro and dejellied with 2% cysteine in 1/10× Modified Barth’s Solution (MBS; Winklbauer, 1988) with the pH adjusted to 8. Embryos were incubated in 1/10× MBS and staged according to Nieuwkoop and Faber (1967). Four-cell-stage embryos in 3% Ficoll solution were microinjected vegetally into all blastomeres. XGulp1 morpholino antisense nucleotide (5′-TTT​GTA​TCA​GAC​CAC​TGC​ACT​CCT​G-3′; GeneTools) was injected at 30 ng per blastomere. To observe vegetal rotation in explants, the blastocoel roof was removed from stage 10 embryos in 1× MBS, and mid-sagittal slices were dissected from the vegetal cell masses. Slices were placed in dishes coated with BSA, secured under glass coverslips, and kept in 1× MBS for 1 h. Explants or whole embryos were fixed in 4% formaldehyde in 1× MBS, and embryos were then fractured mid-sagittally. For XGulp1 immunostaining, vegetal explants were fixed in 4% paraformaldehyde and permeabilized with 0.1% Triton X-100. Explants were stained with rabbit anti-Gulp1 polyclonal antibody (HPA020587; Sigma-Aldrich) and FITC-conjugated secondary antibody. For live imaging, explants from three sets of experiments including 8 and 15 embryos injected with 200 pg and 900 pg ephrinB1-mCherry, respectively, 7 embryos coinjected with ephrinB1-mCherry/XGulp1-MO, and 9 embryos coinjected with ephrinB1-mCherry/XGulp1-MO/XGulp1-GFP were examined by confocal microscopy using a Leica TCS SP8 system.

### Statistical analysis

Statistical analysis was performed with GraphPad Prism software (version 5.00; GraphPad Software). Results were reported as mean ± SEM. No statistical method was used to predetermine sample size. Datasets with data points above five were analyzed with the D’Agnostino and Pearson omnibus normality test. Datasets with normal distributions were analyzed with either Student’s *t* tests to compare two conditions or with a one-way ANOVA Tukey’s test to compare multiple conditions. For data with replicates below five, we assumed normal distribution based on the appearance of the data and analyzed with Student’s *t* test.

### Online supplemental material

Fig. S1 and Videos 1, 2, 3, and 4, related to Fig. 1, show that Gulp1 interacts with EphB2 and ephrinB1 during Eph/ephrin trogocytosis. Fig. S2 and Videos 5 and 6, related to Fig. 3, show that Gulp1 PTB domain mediates its interaction with EphB2 and Gulp1ΔC functions as a dominant-negative form of Gulp1 and blocks EphB2/ephrinB1 trogocytosis. Fig. S3, related to Fig. 4, shows that Tiam2 cooperates with Gulp1 to facilitate Eph/ephrin trogocytosis. Fig. S4, related to Fig. 5, shows that dynamin is necessary for Gulp1 to regulate Eph/ephrin trogocytosis. Videos 7 and 8, related to Fig. 6, show that Gulp1 is required for EphB2/ephrinB1-mediated cell disengagement. Fig. S5, related to Fig. 7, shows that Gulp1 is involved in the movement of vegetal rotation in *Xenopus* gastrula.

## Supplementary Material

Supplemental Materials (PDF)

Video 1

Video 2

Video 3

Video 4

Video 5

Video 6

Video 7

Video 8
