# Supplementary material for: Gulp1 controls Eph/ephrin trogocytosis and is important for cell rearrangements during development
Source: J Cell Biol. 2019 Aug 13;218(10):3455–71. doi: 10.1083/jcb.201901032 (PMC6781437; doi:10.1083/jcb.201901032)
Supplement: Supplemental Materials (PDF) [file JCB_201901032_sm.pdf]

Supplemental material

Gong et al., https://doi.org/10.1083/jcb.201901032

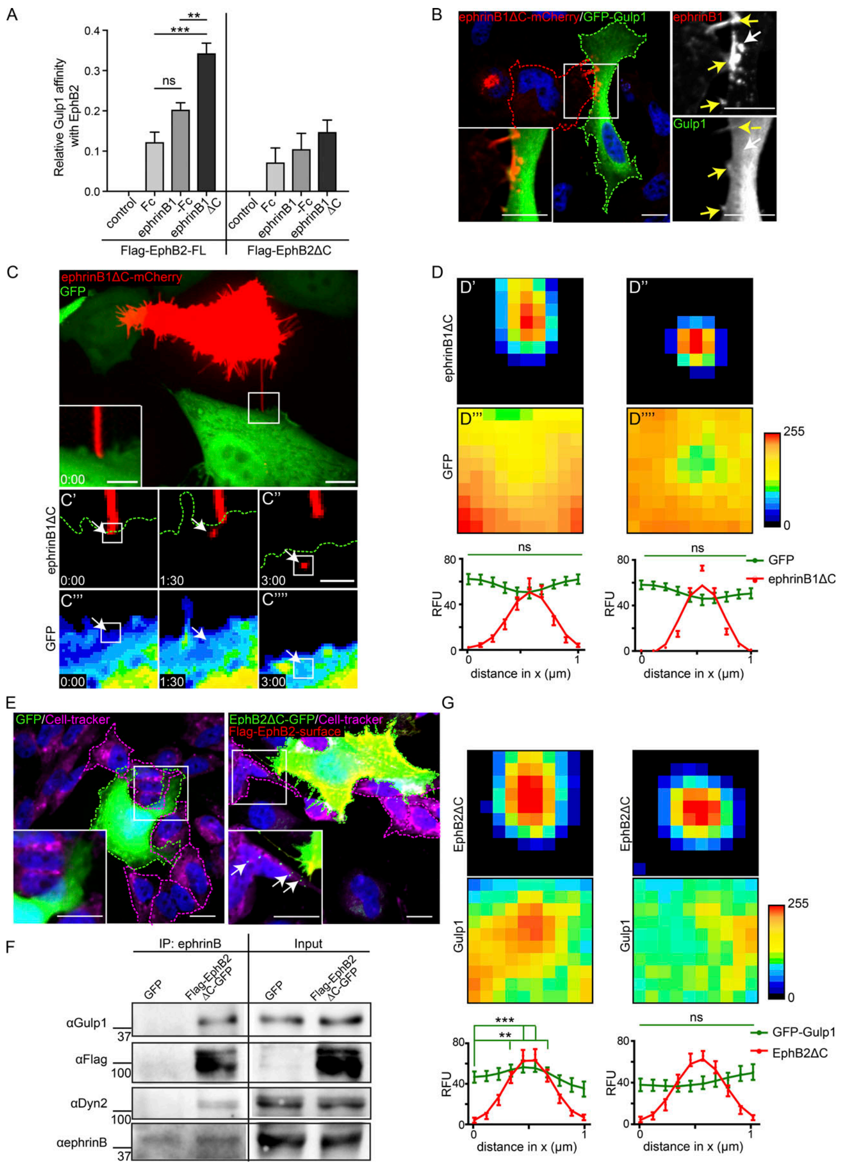

Figure S1. **Gulp1 interacts with EphB2 and ephrinB1 during Eph/ephrin trogocytosis. (A)** Quantification showing the relative interaction intensity between Gulp1 and EphB2 under stated conditions for experiment described in Fig. 1 A. Values shown as mean ± SEM from three independent experiments. **, P < 0.01; ***, P < 0.001; ns, not significant; one-way ANOVA with Tukey's multiple comparisons test. **(B)** Representative images showing localization of Gulp1 at ephrinB1 clusters during forward trogocytosis in HeLa cells. Responder cells (green dashed line) overexpressing Flag-EphB2 with GFP-Gulp1 were cocultured with ephrinB1ΔC-mCherry+ donor cells (red dashed line). White arrow indicates internalized vesicles; yellow arrows indicate surface clusters. Scale bar, 10 µm. **(C)** Representative images from live imaging of forward trogocytosis in HeLa cells. EphrinB1ΔC-mCherry+ donor cells were cocultured with responder cells expressing untagged EphB2-FL and GFP. Middle row: green dashed lines indicate the responder cell outline. Bottom rows show time course at time of contact and scission. Arrows indicate ephrinB1ΔC cluster formation and subsequent vesicle internalization. GFP images pseudo-colored as heat maps (bottom row). Scale bars, 10 µm (top panel), 5 µm (inset), and 2 µm (time lapse images). Elapsed time shown as min:s. **(D)** Average fluorescent intensities at contact sites of cluster formation (left panels) and subsequent vesicles (right panels) from time-lapse imaging of cocultures as described in C. Data displayed as heat maps of average intensity calculated over every event, with donor contact sites for each set aligned to top and center, all images normalized to their respective background signal. Graphs show mean ± SEM of RFU changes through the central four pixels for the x axis. Data acquired from 32 events from 11 cells over two independent experiments. ns, not significant; one-way ANOVA with Bonferroni's multiple comparisons test performed on GFP signal. **(E)** Representative images showing reverse trogocytosis in SKN cells (magenta dashed line, labeled with Cell-tracker) cocultured with EphB2ΔC-GFP+ donor cells (right panels, green dashed line), but not control GFP+ cells (left panels, green dashed line). Internalized EphB2ΔC vesicles in SKN cells were detected as green puncta void of surface Flag-antibody labeling (arrows). Scale bars, 10 µm. **(F)** Validation of the interaction between endogenous Gulp1 and ephrinBs in SKN cells under reverse trogocytosis conditions. SKN cells were first cocultured with either control GFP+ or EphB2ΔC-GFP+ HeLa cells for 30 min, and cell lysates were then subjected to immunoprecipitation by anti-ephrinB antibodies. **(G)** Average fluorescent intensities at contact sites of cluster formation (left panels) and subsequent vesicles (right panels) from time-lapse imaging of reverse trogocytosis in HeLa cells. EphB2ΔC-mCherry+ donor cells were cocultured with responder cells expressing full-length HA-ephrinB1 and GFP-Gulp1. Data displayed as heat maps of the average intensity calculated over every event, with donor contact sites for each set aligned to top and center, all images normalized to their respective background signal. Graphs show mean ± SEM of RFU changes through the central four pixels for the x axis. Data acquired from 28 events from eight cells over two independent experiments. **, P < 0.01; ***, P < 0.001; one-way ANOVA with Bonferroni's multiple comparisons test performed on GFP-Gulp1 signal.

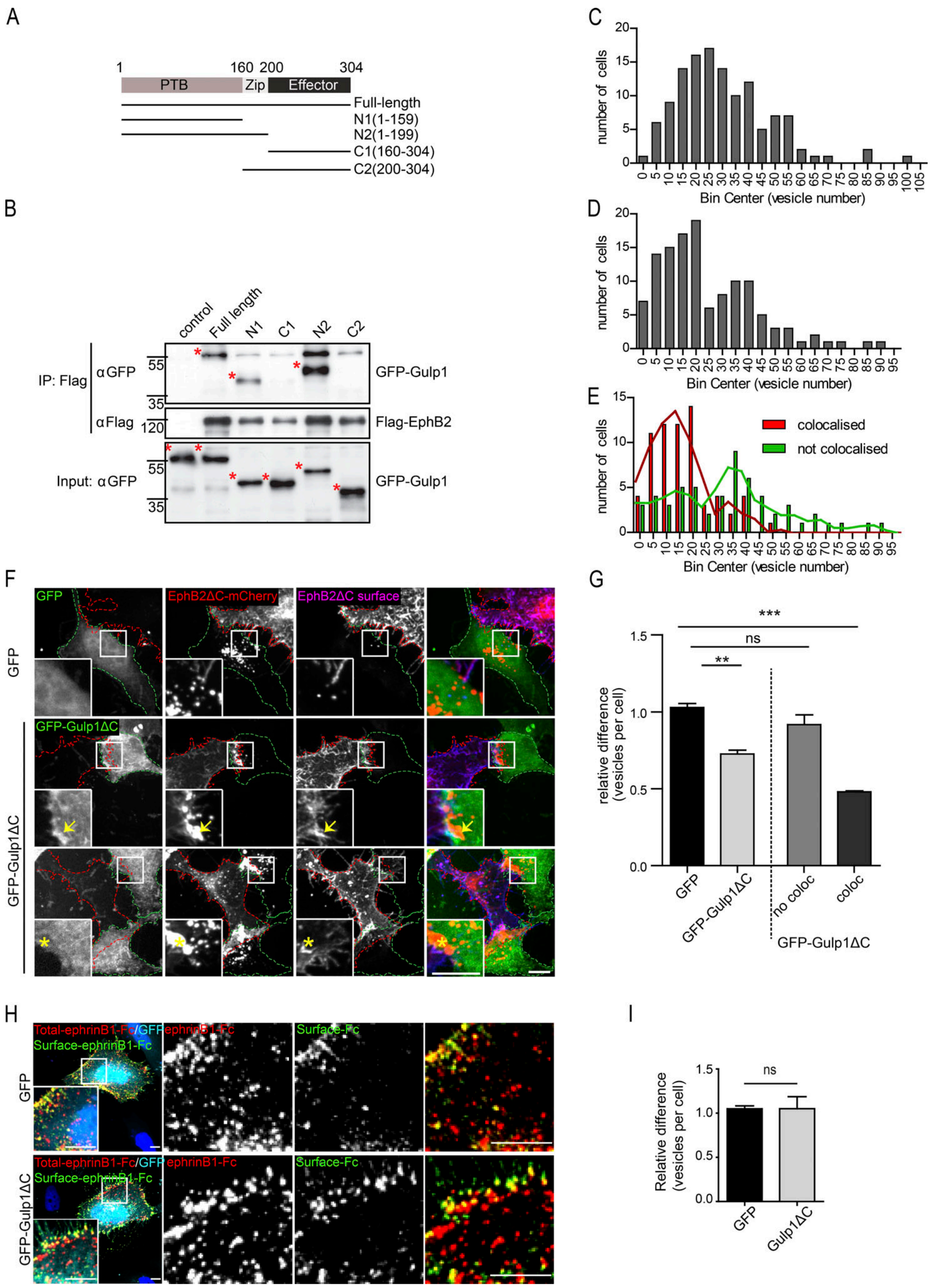

Figure S2. **Gulp1ΔC mediates the interaction of Gulp1 with EphB2 and functions as a dominant-negative form to block EphB2/ephrinB1 trogocytosis.** **(A)** Schematic diagram of Gulp1 truncation constructs. **(B)** Coimmunoprecipitation and Western blots showing interactions of full-length Flag-EphB2 with either full-length or indicated truncations of GFP-Gulp1. Asterisks mark the Gulp1 truncation products. **(C–E)** Histograms showing frequency distribution of vesicle uptake in cells for the experiment described in Fig. 3, B and C. Vesicle numbers separated into bins of five. **(C and D)** Responder cells express full-length EphB2 and either GFP (control; C) or GFP-Gulp1ΔC (dominant-negative; D). **(E)** Two distinct populations of responder cells overexpressing GFP-Gulp1ΔC form when cells are separated into those where Gulp1ΔC colocalizes with the EphB2/ephrinB1 clusters (red bars/curve), and where no colocalization was observed (green bars/curve). n = 125 and 122 responder cells from four independent experiments, respectively. **(F and G)** Representative images (F) and quantification (G) showing Gulp1ΔC blocks reverse trogocytosis when enriched at EphB2/ephrinB1 clusters. Internalized vesicles detected by comparing total EphB2ΔC signal to surface signal. Arrows indicate GFP-Gulp1ΔC enrichment at EphB2/ephrinB1 clusters, and asterisks indicate no GFP-Gulp1ΔC enrichment at EphB2/ephrinB1 clusters. Scale bars, 20 µm. Relative value of vesicle number per cell shown as mean ± SEM (n = 4 independent experiments, 21–24 responder cells per condition per experiment). Data normalized to median GFP value per experiment. The right two columns show the GFP-Gulp1ΔC condition separated into cells that show either GFP-Gulp1ΔC and EphB2ΔC colocalization or not. **, $P < 0.05$; ***, $P < 0.001$; ns, not significant; one-way ANOVA with Dunnett's post hoc test. **(H and I)** Representative images (H) and quantification (I) showing GFP-Gulp1ΔC has no effect on ephrinB1-Fc–induced endocytosis in HeLa cells overexpressing full-length EphB2. Cells were stimulated with fluorescently labeled, preclustered ephrinB1-Fc, fixed without permeabilization, and immunostained against Fc (surface ephrinB1-Fc). Cells were imaged and manually scored for internalized vesicles. Results shown as mean ± SEM (n = 3 independent experiments, 12–23 cells per condition per experiment). Data normalized to median GFP value per experiment. ns, not significant; two-tailed unpaired t test.

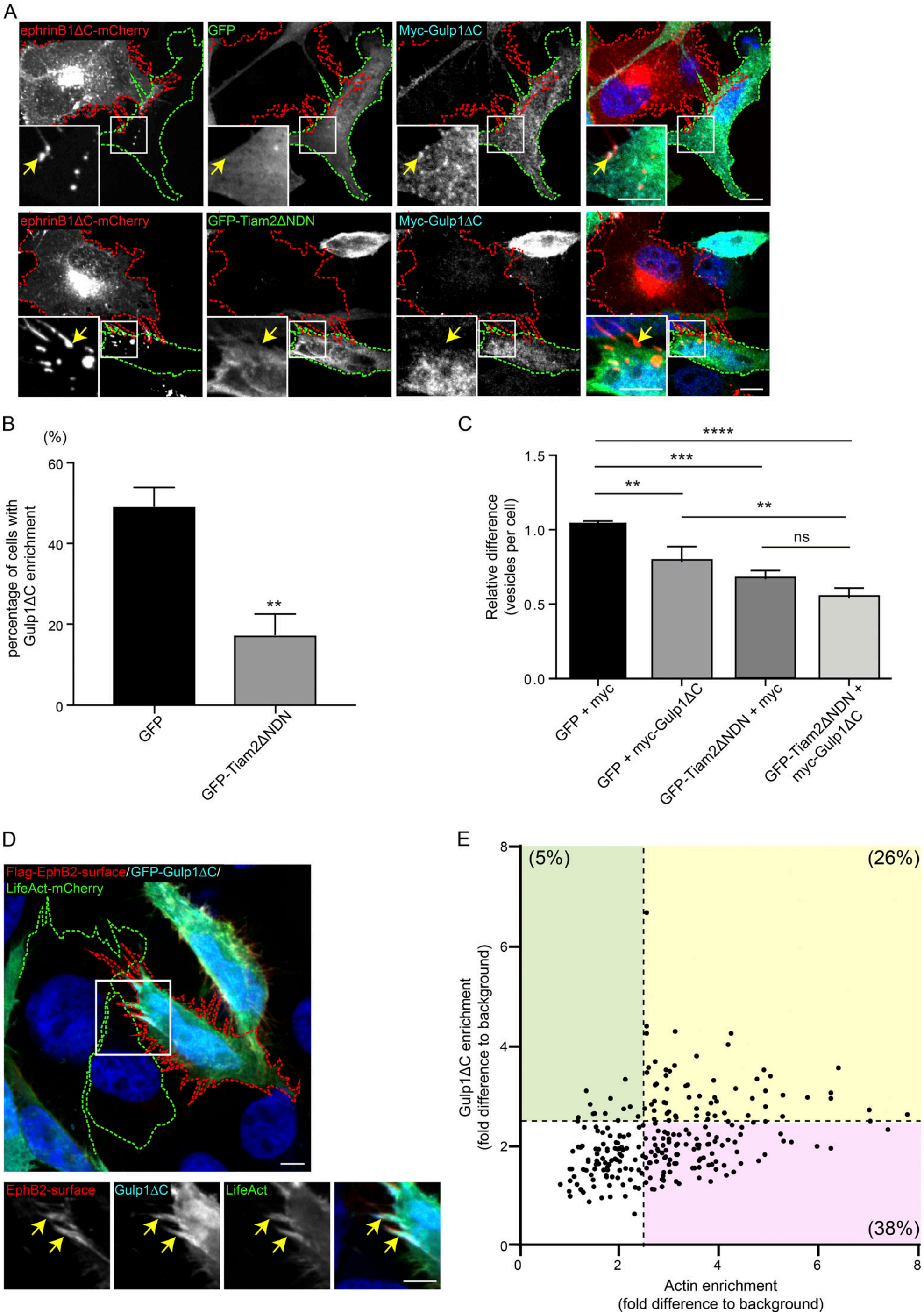

Figure S3. **Tiam2 cooperates with Gulp1 to facilitate Eph/ephrin trogocytosis. (A and B)** Representative images (A) and quantification (B) showing dominant-negative Tiam2 (Tiam2ΔNDN) reduces enrichment of Gulp1ΔC at EphB2/ephrinB1 clusters in HeLa cells. Responder cells (green dashed line) overexpressing Flag-EphB2 and myc-Gulp1ΔC together with either GFP-Tiam2ΔNDN or GFP as control were cocultured with ephrinB1ΔC-mCherry⁺ donor cells (red dashed line). Arrows indicate ephrinB1ΔC clusters. Scale bars, 10 µm. Percentage of cells with myc-Gulp1ΔC enrichment at ephrinB1ΔC clusters shown as mean ± SEM (n = 3 independent experiments, 16–27 responder cells per condition per experiment). **, P = 0.0013; two-tailed unpaired t test. **(C)** Quantification of coculture assay showing dominant-negative Tiam2 and Gulp1ΔC have no synergistic effect in reducing forward EphB2/ephrinB1 trogocytosis. Relative values of vesicle numbers per cell (normalized to median GFP/myc value per experiment) shown as mean ± SEM (n = 3 independent experiments, 16–36 responder cells per condition per experiment). **, P < 0.01; ***, P < 0.001; ****, P < 0.0001; ns, not significant; one-way ANOVA with Tukey's multiple comparisons test. **(D)** Representative images showing Gulp1ΔC does not interfere with actin polymerization at EphB2/ephrinB1 clusters. Arrows in enlarged insets show co-localizaton and enrichment of LifeAct and Gulp1ΔC at the same EphB2 clusters. Scale bars, 10 µm. **(E)** Quantification of LifeAct and Gulp1ΔC enrichment at EphB2 clusters for experiment described in D. Maximum fluorescence intensities of LifeAct and GFP-Gulp1ΔC within each EphB2 cluster regions of interest were measured and normalized to background (value from a region in the same responder cell without EphB2 clusters or vesicles). n = 4 independent experiments, 8–15 cells per experiment; each point represents an independent cluster. Threshold to indicate enrichment was set to 2.5 times that of background.

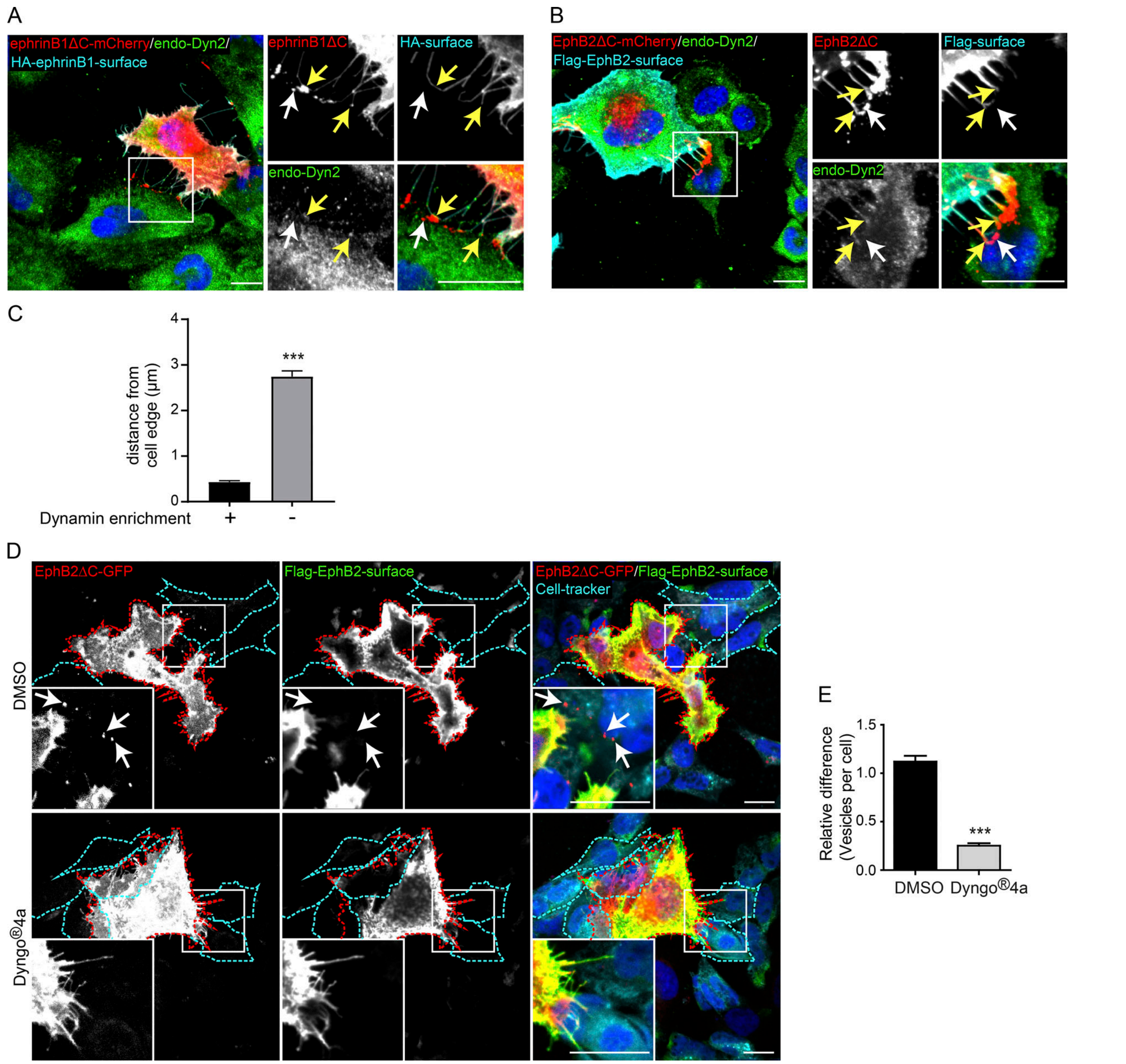

Figure S4. **Dynamin is required for Eph/ephrin trogocytosis. (A)** Representative images showing endogenous Dyn2 enriches at ephrinB1 clusters during forward trogocytosis in U251 cells. U251 cells were cocultured with HA-ephrinB1ΔC-mCherry+ HeLa donor cells. Cells were fixed and stained against HA without permeablization (surface ephrinB1 clusters) and then permeablized and stained against endogenous Dyn2. White arrows indicate internalized vesicles; yellow arrows indicate surface clusters. **(B)** Representative images showing endogenous Dyn2 enriches at EphB2 clusters during reverse trogocytosis in SKN cells. SKN cells were cocultured with Flag-EphB2ΔC-mCherry+ HeLa donor cells. Cells were fixed and stained against Flag without permeablization (surface EphB2 clusters) and then permeablized and stained against endogenous dynamins. White arrows indicate internalized vesicles; yellow arrows indicate surface clusters. **(C)** Quantification of the distance from cell edge for ephrinB1ΔC clusters with and without dynamin enrichment. The distance of each ephrinB1ΔC cluster from cell surface was measured for data from Fig. 5 E. ***, P < 0.0001; two-tailed unpaired *t* test. **(D and E)** Representative images (D) and quantification (E) showing inhibition of dynamin activity blocks reverse trogocytosis in SKN cells. SKN cells (cyan dashed line, labeled with Cell-tracker) treated with either DMSO or Dyngo-4a before being cocultured with Flag-EphB2ΔC-GFP+ donor HeLa cells (red dashed line). Cells were fixed and stained against Flag without permeablization (surface EphB2 clusters). Arrows indicate internalized EphB2ΔC vesicles. Quantification results shown as mean ± SEM (*n* = 3 independent experiments, 29–34 responder cells per condition per experiment, normalized to median DMSO value per experiment). ***, P = 0.0002; two-tailed unpaired *t* test. Scale bars, 10 µm.

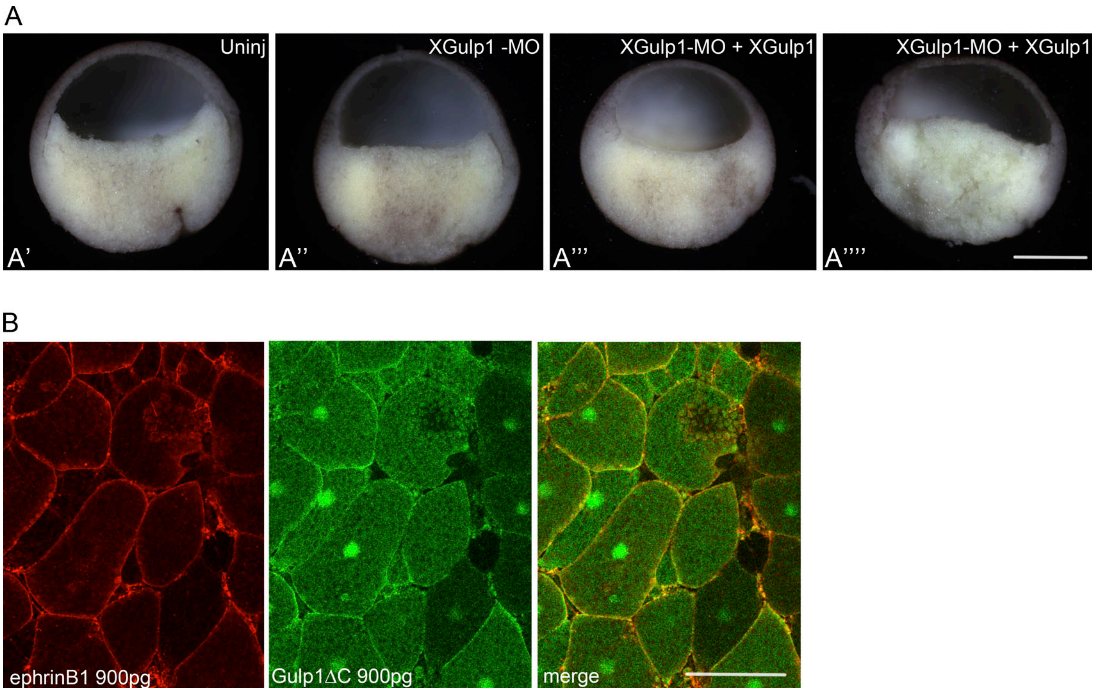

Figure S5. **Gulp1 is involved in cell rearrangement during the *Xenopus* gastrulation process. (A)** Mid-sagittal fractures of *Xenopus* early gastrulae. Compared with uninjected (A′) and XGulp1-MO (A″) injected embryos, respectively, the curvature of the blastocoel floor is normal (concave) in 20 of 29 (A′′′) but reversed (convex) in 9 (A′′′′) XGulp1-MO/XGulp1 coinjected embryos. The abnormal convex curvature may be due to ongoing vegetal rotation and a lack of mesodermal migration that normally parallels vegetal rotation, similar to the behavior of explanted vegetal slices (see Fig. 7 D). These embryos were not included in the curvature measurements. Scale bar, 500 µm. **(B)** Cells in vegetal slice explants after coinjection of ephrinB1-mCherry (red) and mouse Gulp1ΔC mRNA (green). Scale bars, 100 µm.

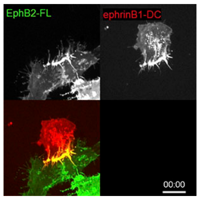

Video 1. **Representative video for Fig. 1 B showing forward trogocytosis when responder EphB2-GFP⁺ HeLa cells were cocultured with donor ephrinB1ΔC-mCherry⁺ HeLa cells.** Scale bar, 10 µm. Elapsed time shown as min:s. Arrow indicates the formation of EphB2/ephrinB1 clusters upon cell–cell contact and subsequent trogocytosis of ephrinB1 into EphB2⁺ cells.

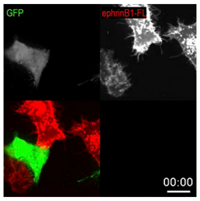

Video 2. **Representative video showing no cluster formation and no trogocytosis when GFP⁺ HeLa cells were cocultured with ephrinB1-mCherry⁺ HeLa cells.** Scale bar, 10 µm. Elapsed time shown as min:s. Related to Fig. 1.

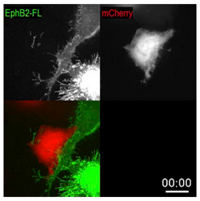

Video 3. **Representative video showing no cluster formation and no trogocytosis when EphB2-YFP⁺ HeLa cells were co-cultured with mCherry⁺ HeLa cells.** Scale bar, 10 µm. Elapsed time shown as min:s. Related to Fig. 1.

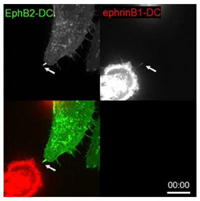

Video 4. **Representative video showing no trogocytosis was detected when EphB2ΔC-GFP⁺ HeLa cells were cocultured with ephrinB1ΔC-mCherry⁺ HeLa cells.** Scale bar, 10 µm. Elapsed time shown as min:s. Arrows indicate the formation of EphB2/ephrinB1 clusters upon cell–cell contact. Related to Fig. 1.

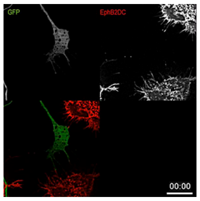

Video 5. **EphB2ΔC trogocytosis into primary neurons.** Representative video for Fig. 3 D showing reverse trogocytois of EphB2ΔC clusters from EphB2ΔC-mCherry⁺ donor HeLa cells into primary mouse cortical neurons expressing GFP. Scale bar, 10 µm. Elapsed time shown as min:s.

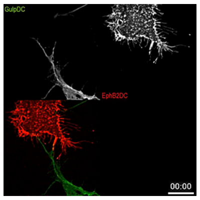

Video 6. **Gulp1ΔC inhibits EphB2ΔC trogocytosis into primary neurons.** Representative video for Fig. 3 D showing inhibition of reverse trogocytosis of EphB2ΔC clusters from EphB2ΔC-mCherry⁺ donor HeLa cells into primary mouse cortical neurons expressing GFP-Gulp1ΔC. Scale bar, 10 µm. Elapsed time shown as min:s.

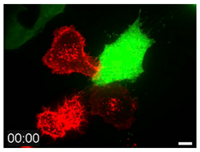

Video 7. **EphB2/ephrinB1-mediated cell disengagement in HeLa cells.** Representative video for Fig. 6 A showing HeLa cells expressing EphB2 and GFP (green) were cocultured with ephrinB1ΔC-mCherry⁺ cells (red) and imaged live. Scale bar, 10 µm. Elapsed time shown as min:s.

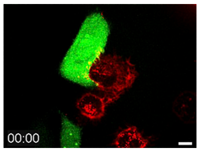

Video 8. **Gulp1ΔC inhibits EphB2/ephrinB1-mediated cell disengagement in HeLa cells.** Representative video for Fig. 6 B showing HeLa cells expressing EphB2 and GFP-Gulp1ΔC (green) were cocultured with ephrinB1ΔC-mCherry⁺ cells (red) and imaged live. Scale bar, 10 µm. Elapsed time shown as min:s.
